# A flexible, allosteric loop regulates protein activity and rewires electrostatics

**DOI:** 10.1002/pro.70315

**Published:** 2025-09-24

**Authors:** Darex J. Vera‐Rodríguez, Paul J. Sapienza, Konstantin I. Popov, Andrew L. Lee

**Affiliations:** ^1^ Department of Biochemistry and Biophysics, School of Medicine University of North Carolina at Chapel Hill Chapel Hill North Carolina USA; ^2^ Division of Chemical Biology and Medicinal Chemistry, Eshelman School of Pharmacy University of North Carolina at Chapel Hill Chapel Hill North Carolina USA

**Keywords:** allostery, loops, NMR, paramagnetic relaxation enhancement, protein dynamics, protein electrostatics

## Abstract

Allostery is a key driver of protein function and behavior in biological systems. Historically, allosteric regulation has been attributed to conformational and dynamic changes, mostly derived from well‐structured regions of proteins. While the regulatory contributions of specific unstructured elements, such as catalytic loops near the active site, have been widely characterized, the role of distal, flexible loops remains poorly understood. Here, we investigate the allosteric protein chorismate mutase (CM), a homodimeric enzyme critical for the biosynthesis of aromatic amino acids. Although CM is differentially regulated by tryptophan and tyrosine via a shared pocket over 25 Å from the active site, their near‐identical NMR spectra suggest that alternative mechanisms may explain TrpCM and TyrCM's distinct functional landscapes. We demonstrate that a mutation within a structurally invisible and highly flexible loop, loop 11–12, located far from the active site, drastically alters CM's activity landscape. Using paramagnetic labeling of the loop, we show that loop 11–12 undergoes transient excursions toward the active site, but only in the presence of the activator Trp, which binds over 20 Å away. Furthermore, employing a novel NMR approach, we show that loop 11–12 modulates CM's electrostatics, potentially influencing charge distribution to provide another control of enzymatic activity. Our findings support a sophisticated allosteric process in which a flexible, distal loop is functionally coupled to both the effector binding region and the active site. This mechanism provides new insights into the diverse ways proteins achieve allosteric regulation and may contribute to understanding flexible regions in other allosteric systems.

## 1 INTRODUCTION

Allostery is a fundamental biological phenomenon characterized by signal transmission between distal sites within a macromolecule to modulate function. Traditionally, models of allostery have focused on discrete conformational changes as the primary mechanism by which ligand binding alters protein activity (Koshland et al., 1966; Monod et al., 1965). More recently, growing evidence has suggested that protein dynamics play a crucial role in allosteric regulation (Bonin et al., 2022; Cooper & Dryden, 1984; Hilser et al., 2012; Kern & Zuiderweg, 2003; Motlagh et al., 2014; Petit et al., 2009; Popovych et al., 2006; Tzeng & Kalodimos, 2012). Protein dynamics are typically identified by characterizing motions in structured regions of the protein, but can also be captured by examining large, flexible regions—such as loops. Catalytic or active site loops, which often transition between “open” or “closed” conformations, have been widely characterized as drivers of protein activity (Papaleo et al., 2016). However, in some instances, activity can be modulated by flexible loops distal to the active site (Barkho et al., 2013; Doucet et al., 2009; Hedstrom et al., 1992; Josephine et al., 2010; Mace et al., 1995). While it is unclear how distal flexibility might influence activity, such systems should ultimately provide new insights into how dynamics can factor into allosteric regulation. The challenge, experimentally, is that the dynamics of flexible elements in proteins can be difficult to observe and characterize, and the connectivity to the distal active site may not be clear. Overcoming this challenge would provide deeper insight into how flexible regions impact activity through allosteric mechanisms, expanding our conception of allosterism and underscoring the role of dynamics in protein function.

**FIGURE 1:**
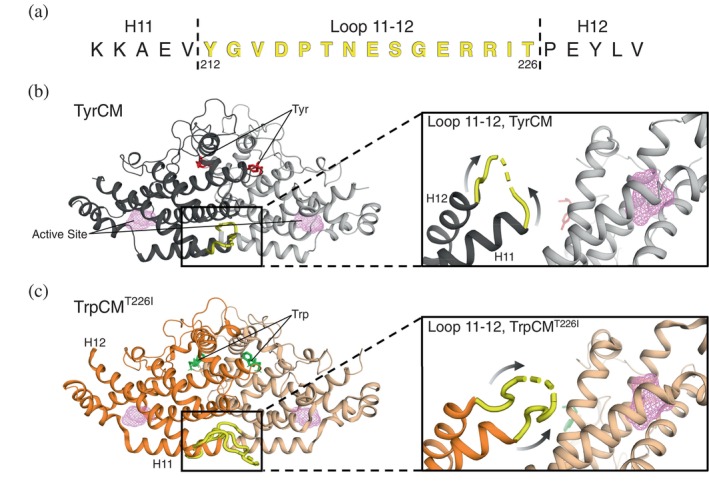
Structural features of active and inhibited forms of chorismate mutase (CM). (a) Amino acid sequence of loop 11–12 (yellow), with adjacent portions of helix 11 (H11) and helix 12 (H12) highlighted. The N‐ and C‐termini of loop 11–12 are labeled with their corresponding residue numbers for reference. (b) The wild‐type CM structure is shown bound to tyrosine (Tyr, red), and (c) the T226I CM variant is shown bound to tryptophan (Trp, green). In both structures, the active site region is highlighted in magenta mesh. CM homodimer's subunits are distinguished using different shades of the same color. The zoomed‐in regions highlight loop 11–12's edges (yellow) structural positioning. Residue V214 serves as a reference point for the position of loop 11–12 in both structures, as it represents the last visible residue at the N‐terminal end of the loop in TyrCM. Residues 215–223 and 218–221 are not resolved in the TyrCM and TrpCM^T226I^ crystal structures, respectively. In TrpCM^T226I^, residues 214–217 and 222–224 displayed elevated B‐factors of at least 50 Å^2^ and up to 100 Å^2^. PDB entries: 2CSM (TyrCM) and 1CSM (TrpCM^T226I^).

To provide insights into these and other questions, we have turned to chorismate mutase (CM), which is an ideal model for studying classical allostery. This 60 kDa homodimeric enzyme plays a pivotal role in the Shikimate pathway, determining flux for aromatic amino acid synthesis via feedback regulation. CM's activity is regulated by the binding of tryptophan (activator) and tyrosine (inhibitor) more than 25 Å from the active site, inducing a heterotropic allosteric effect (Schnappauf, Krappmann, & Braus, 1998). Interestingly, while TrpCM and TyrCM exhibit markedly different activity profiles, our previous study using ^1^H‐^13^C NMR have demonstrated that both the tryptophan‐ and tyrosine‐bound states primarily populate the same ground, T‐state, with only minimal excursions to an excited, R‐state observed in TrpCM (Sapienza et al., 2023). This excited state corresponded well with the conformation derived from the T226I mutant bound to tryptophan, TrpCM^T226I^, a constitutively activated CM variant that remains adopts the R‐state (Sapienza et al., 2023; Schmidheini et al., 1989; Schmidheini et al., 1990). Notably, this T226I modification is located at the C‐terminal end of a flexible, largely unobserved loop between helices 11 and 12, referred to here as loop 11–12 (Xue et al., 1994). Previous studies have identified this loop as an important region for CM allosteric regulation (Helmstaedt et al., 2002). Briefly, they demonstrated that exchanging yeast CM's loop 11–12 with analog loops from other fungal CMs with similar structural alignment led to altered enzyme regulatory responses. While that work revealed the functional involvement of loop 11–12, the precise mechanism by which it mediates these regulatory effects remains unclear. The visible segments of loop 11–12 in the TyrCM and TrpCM^T226I^ crystal structures suggest that it adopts different orientations with additional flexibility in the middle depending on the allosteric state (Figure 1) (Strater et al., 1996; Xue et al., 1994). However, the absence of a wild‐type TrpCM structure and the lack of electron density from the central portion of the loop in all available crystal forms limit our ability to interpret the conformational variations accessed by loop 11–12.

Herein, we investigate the involvement of loop 11–12's flexibility in modulating protein activity and facilitating allosteric communication in different CM states. We show that loop 11–12, despite its distance from the active site, is important for maintaining enzymatic activity, underscoring its regulatory significance in CM. Specifically, the activity alterations induced by a single‐site mutation in this loop occur without major conformational rearrangements in the overall protein, suggesting that its functional influence is mediated through more subtle and transient structural effects. By employing PRE NMR spectroscopy, we reveal that the conformational variability of loop 11–12 is modulated by the identity of the bound effector. We show that loop 11–12 undergoes a distinctive reorientation toward the active site entrance that is only observed when the activator Trp is bound to the protein. Moreover, our findings offer insights into an intricate electrostatic rearrangement mediated by loop 11–12, which may be crucial for maintaining the proper charge distribution required for CM's catalytic function. We propose a model where loop 11–12, through its intrinsic flexibility, promotes electrostatic remodeling of the active site region to preserve CM's catalytic activity. Collectively, these results highlight a rare instance of allosteric communication where a distal, flexible loop interacts with key regions of the protein to orchestrate long‐range signaling and regulate protein function.

## 2 RESULTS

### 2.1 A single‐point mutation within a flexible, distant loop regulates CM's activity

Loop 11–12 (residues 212–226) in CM was previously mentioned as being potentially “connected” with the active site of the enzyme, despite its overall physical separation in the structure (Lin et al., 1998). The basis for this connection—direct or indirect—is unclear due to the loss of electron density in much of the loop (Figure 1). While the change in loop conformation is evident from its visible edges, the degree of flexibility in the main body of the loop is not well characterized. As a starting point, we sought to determine the extent of loop 11–12 flexibility under solution conditions using NMR spectroscopy. Backbone amide triple‐resonance assignments revealed the disappearance of more than half of the resonances corresponding to loop 11–12 in both the apo and TrpCM forms of the protein, indicative of significant signal line broadening from dynamics or peak intensity dilution due to a distribution of conformations sampled by the loop (Figure S1). Notably, signals from residues 214–220 of the N‐terminal segment of loop 11–12 were missing from the HSQC spectra in both states. Previous studies proposed, through analysis of MD simulations, that D215 within this N‐terminal region may facilitate polar contacts between loop 11–12 and the active site region (Lin et al., 1998). Considering the evident flexibility of this loop and its hypothesized role in structurally communicating with the active site, we aimed to investigate whether specific residues within loop 11–12 contribute to the regulation of protein activity.

**FIGURE 2:**
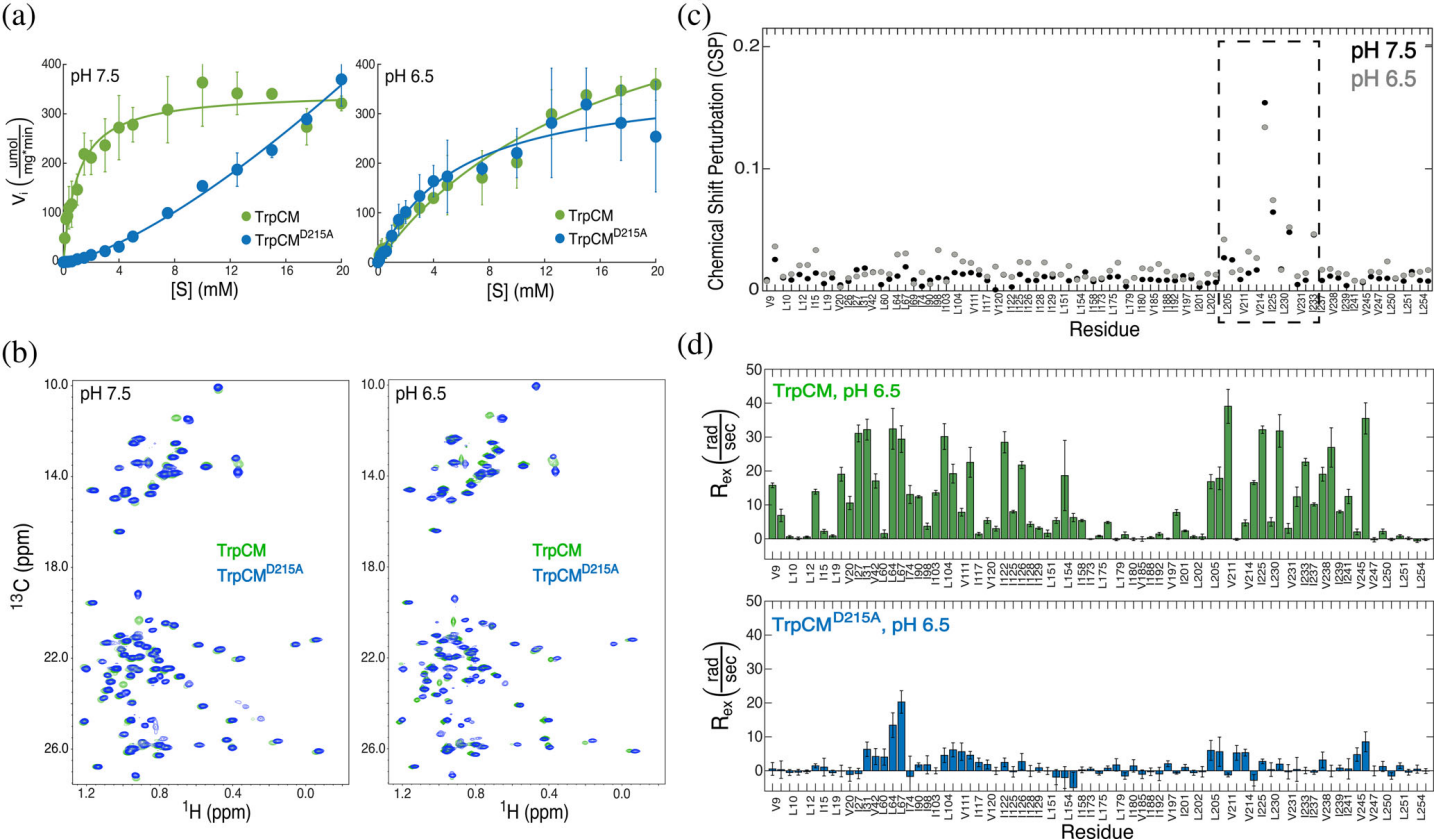
Enzymatic activity and NMR spectroscopic analyses of TrpCM and TrpCM^D215A^. (a) Enzyme kinetics of TrpCM (green) and TrpCM^D215A^ (blue) measured at pH 7.5 (left panel) and pH 6.5 (right panel). Data points represent averages from triplicate measurements. (b) Isoleucine, leucine, and valine (ILV) ^1^H‐^13^C HMQC spectra of TrpCM (green) and TrpCM^D215A^ (blue) recorded at pH 7.5 (left panel) and pH 6.5 (right panel). (c) Chemical shift perturbation (CSP) analyses from ILV ^1^H‐^13^C HMQC spectra comparing TrpCM and TrpCM^D215A^ at pH 7.5 (black) and pH 6.5 (gray). The dashed box highlights regions in the vicinity of loop 11–12, where the most significant CSPs occur, indicating structural changes likely in response to the mutation. (d) ^1^H CPMG relaxation dispersion analysis of TrpCM (upper panel) and TrpCM^D215A^ (lower panel), presented as *R*_ex_ values plotted against ILV residues.

To pinpoint the role of individual residues in loop 11–12 to CM function, we evaluated the enzymatic activity of the D215A CM mutant. A significant loss of CM activity was observed, even in the presence of the activator tryptophan, for which a marked decrease in substrate binding affinity is evident (Figure 2a). Interestingly, this D215‐inhibitory effect is pH‐dependent: at pH 7.5, TrpCM^D215A^ activity is dramatically reduced, yet at pH 6.5, activity is restored to wild‐type levels (Figure 2a). Additionally, unlike the hyperbolic kinetics observed in the wild‐type enzyme, TrpCM^D215A^ at pH 7.5 displays a sigmoidal curve. This transition from a hyperbolic to a sigmoidal‐shaped activity curve suggests that the D215A mutation introduces a cooperative effect, albeit with reduced substrate binding, into the enzyme's function as a potential compensatory mechanism. This effect is unique to D215A, as a similar mutation (E219A) at the only other charged residue near the middle of loop 11–12 did not affect substrate affinity or the activity curve shape (Figure S2). The inhibition by D215A of protein activity suggests that loop 11–12 functions as an allosteric regulatory element, an uncommon feature for distal, flexible loops of a protein. Furthermore, the pH‐reversibility of this effect suggests a mechanistic pathway in which CM modulates the ionic environment of critical regions in the protein to maintain its catalytic properties.

### 2.2 Loop 11–12's regulatory role is not based on global conformational changes or switching dynamics

The classic allosteric models hold that proteins adjust their activity levels in response to a conformational change. Hence, proteins adopt a “tense” (T‐state) conformation in their less‐active form and a “relaxed” (R‐state) conformation in their more active form (Koshland et al., 1966; Monod et al., 1965). Our previous work using methyl NMR spectroscopy revealed pronounced and easily recognized peak‐position differences between CM's T‐ and R‐states (Sapienza et al., 2023). To assess whether D215A caused any structural changes in the protein that could contribute to CM's activity discrepancy, we collected ILV ^1^H‐^13^C HMQC spectra and monitored chemical shift perturbations (CSPs) at both pH 6.5 and 7.5. Figure 2b,c shows no significant CSPs on residues distant from loop 11–12 when comparing TrpCM^D215A^ with TrpCM at either pH. The only notable CSPs occurred at residues L205, V211, V214, I225, L230, and I233 (Figure 2b,c), located in the vicinity of loop 11–12's N‐ and C‐termini, and for which discrepancies were anticipated due to their proximity to the mutation. Although the D215A mutation suppresses CM's activity at pH 7.5, it does not induce major conformational changes, and the protein largely remains in its ground, T‐state. The modest structural differences between TrpCM^D215A^ and TrpCM at pH 7.5 imply that the allosteric inhibition mechanism of TrpCM^D215A^ is more sophisticated and cannot be attributed to conformational changes. Overall, CM structural features do not correlate with activity.

We recently showed that while TrpCM predominantly adopts a T conformation, it dynamically samples the R conformation (Sapienza et al., 2023). Although TrpCM populates the R‐state to less than 10%, the possibility remains that dynamic excursions into the R‐state may underlie its high catalytic activity. To test if the behavior of TrpCM^D215A^ is governed by such conformational switching dynamics, we employed ^1^H Carr‐Purcell‐Meiboom‐Gill (CPMG) NMR experiments to assess the dynamic profile of TrpCM^D215A^ relative to TrpCM. Figure 2d shows that the D215A mutation quenches conformational exchange across almost the entire protein. Strikingly, this dynamics suppression was observed for TrpCM^D215A^ at pH 6.5, where the protein's activity is very similar to the wild‐type form. While we previously determined that a large group of residues in wild‐type TrpCM undergo a concerted T‐to‐R conformational exchange at a rate of *k*_ex_ = 670 s^−1^ (Sapienza et al., 2023), global fitting attempts for TrpCM^D215A^ were unsuccessful, as the amplitudes of the relaxation dispersion curves were too small to yield reliable fits. This is consistent with the majority of residues in TrpCM^D215A^ exhibiting quenched dynamics, indicative of a loss of conformational exchange. The effector binding region (EBR, residues 42–104) was the only region displaying notable switching, which exhibits a more complex dynamic repertoire that deviates from a simple two‐state conformational exchange (Sapienza et al., 2023). The dynamics‐quenching effect induced by D215A while maintaining its enzymatic function indicates that a dynamic, two‐state conformational switching model is also inconsistent with CM's activity change.

### 2.3 Loop 11–12 dynamics are differentially biased by CM effectors

**FIGURE 3:**
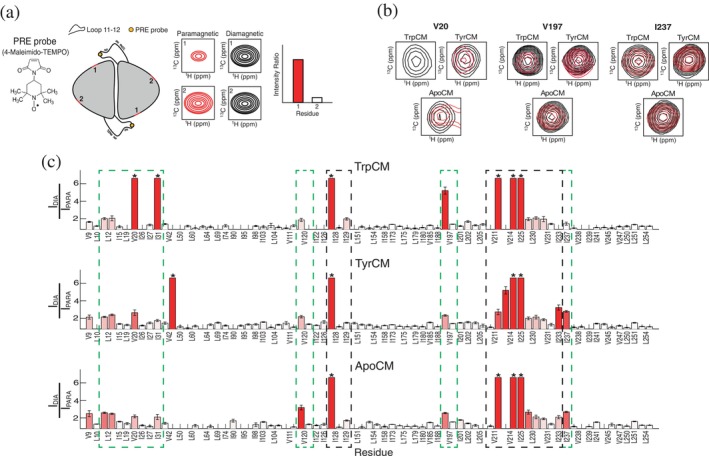
Tracking loop 11–12 structural positioning via PRE NMR spectroscopy. (a) Schematic representation of the PRE experiment. A paramagnetic spin label is introduced into loop 11–12 to monitor its spatial positioning. Regions in proximity to the loop experienced peak broadening due to rapid relaxation induced by the spin label. Intensity ratios were calculated by comparing paramagnetic (oxidized spin label) and diamagnetic (reduced spin label) states. (b) Residue‐specific signal broadening caused by the PRE effect are shown through peak overlays between diamagnetic (black) and paramagnetic (red) forms of TrpCM, TyrCM, and apoCM. (c) PRE profiles for CM methyl residues for TrpCM (upper panel), TyrCM (middle panel), and apoCM (bottom panel). Higher PRE values indicate closer proximity of loop 11–12 to specific regions of the protein, while lower values suggest greater distance. Regions with the most pronounced PRE effects are highlighted with dashed boxes for group 1 (black) and group 2 (green) as described in the main text. Bars are color‐coded using a red gradient to emphasize variations in PRE intensity. Residues marked with an asterisk denote complete signal disappearance due to strong PRE effects. Empty spaces indicate residues for which lack of NMR assignments or resonance overlap prevented analysis. Details of TrpCM residues for which PRE ratios were calculated at 150 mM or 0 mM NaCl to allow for detection of more methyl probes are provided in Table S1.

Explaining how mutation of the flexible loop 11–12 alters CM function is challenging. The single‐structure view fails because D215A appears to be at least 10 Å or further from the active site, as shown in the crystal structures. Adding another state (two‐structure view) to enable an inactive‐to‐active mechanism also fails, as described in the previous section. For these reasons, we considered that loop 11–12 might control CM activity via particular transient excursions affecting the active site. To understand how this might occur, general location information on loop conformational sampling should be highly valuable. Given that we are unable to directly monitor loop 11–12 due to the absence of signal in NMR spectra (Figure S1), we placed a paramagnetic relaxation enhancement (PRE) probe within the loop as a proxy to track regions of the protein in proximity to loop 11–12, effectively mapping its spatial positioning (Figure 3a).

PRE methods have been instrumental in elucidating various aspects of macromolecular behavior, ranging from detecting low‐populated conformations (Baker et al., 2006; Clore et al., 2007; Iwahara & Clore, 2006; Tang et al., 2007) to gaining structural insight into intrinsically disordered proteins (Mateos et al., 2019; Salmon et al., 2010). PRE‐based NMR experiments are unique in that they can detect diverse conformations of flexible structural elements over different timescales, unlike other NMR techniques, which are often limited to identifying only two‐ or three‐state switching behaviors. Placement of a nitroxide spin label in the middle of loop 11–12 (see section 4) allowed detection of PRE effects at nearby NMR‐visible sites in the protein. Because PRE effects of nitroxide probes are observed up to a maximum of 25 Å (Griffith & McConnell, 1966), we used PRE‐induced peak intensity losses to trace loop 11–12 positioning for CM states with different activities.

The basic PRE effect is for the paramagnetic spin label to accelerate relaxation of normal NMR peak signals, reducing 2D peak intensities in NMR spectra when the distance to the spin label is close enough (Figure 3a,b). PREs were observed in side‐chain methyl sites using ^1^H‐^13^C TROSY HMQC spectra, which allows for high sensitivity and greater protein coverage since most CM methyl signals have been assigned (Gorman et al., 2018; Sapienza et al., 2023). Importantly, PREs were measured for three separate allosteric states of CM, according to which effector was bound: apo, Trp, and Tyr. Overall, the observed PREs in all states could be loosely placed into two groups (Figure 3c). Group 1 contains residues proximal to the spin label at position 220 and is therefore structurally proximal to loop 11–12. This includes residues at the loop's stubs (V211, V214, and I225) but also includes a cluster of proximal residues near the loop edges: I128, I129, L230, V231, and I233. The location of this cluster is consistent with loop positioning in the ground, T‐state, as opposed to the R‐state, in which the loop stubs are directed away from this cluster (Figure 1). Indeed, apoCM, TrpCM, and TyrCM show NMR spectra consistent with the T‐state (Sapienza et al., 2023). The second group of PREs, group 2, contains residues that fall outside this proximal range and are therefore likely to arise from dynamic loop excursions. They are L12, V20, I31, V120, V197, and I237 (Figure 3c). They lie predominantly in the outer “tips” of the dimer, away from the dimer interface (except V20) at distances >20 Å from the residues in loop 11–12. More specifically, many of these residues are located in the vicinity of the substrate binding site, suggesting that loop 11–12 can relocate from its “home” location to the exposed surfaces of CM's active site. This distal PRE group can only be explained if loop 11–12 undergoes significant, and perhaps transient, relocation across CM's protein surface.

**FIGURE 4:**
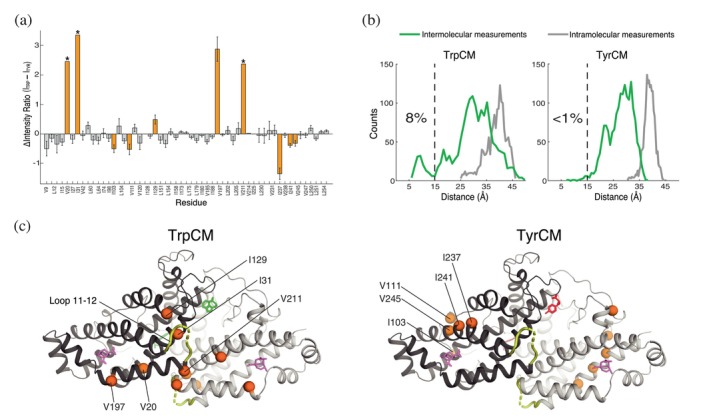
PRE differences and MD simulations suggest intermolecular interactions between loop 11–12 and CM's active site region in TrpCM. (a) Quantified PRE differences (Δ*I* = *I*_TRP_ – *I*_TYR_) between TrpCM and TyrCM for residues with assignments in both CM forms. Positive values (Δ*I* > 0) indicate higher PREs for TrpCM, whereas negative values (Δ*I* < 0) indicate higher PREs for TyrCM.Residues with pronounced PRE differences between TrpCM and TyrCM are highlighted in orange. Asterisks denote residues where full peak bleaching was observed due to close proximity of loop 11–12. (b) MD simulations revealing inter‐ and intra‐promoter differences in loop 11–12 positioning. Distances between loop 11–12 and the active site collected for intermolecular (green) and intramolecular (gray) interactions are shown for TrpCM (left) and TyrCM (right). Distances are plotted against the frequency of occurrence (counts) for each CM form. A dashed line at 15 Å marks the threshold for identifying interactions within this proximity, with the percentage of distances below this threshold indicated for each form. (c) Structural visualization of PRE‐affected regions of TrpCM and TyrCM forms. The orange spheres highlight residues with pronounced PRE differences between TrpCM and TyrCM. Only residues from one subunit are labeled for clarity. The substrate (magenta) is shown solely to reference the active site, as PRE experiments were conducted in the absence of the substrate analog. PDB ID: 2CSM.

A major goal of understanding loop 11–12 flexibility and its influence on CM function is whether it is modulated by allosteric effectors Trp and Tyr. Though the activator, Trp, and the inhibitor, Tyr, share the same binding site more than 20 Å away from loop 11–12's edges, they induce distinctly different loop conformations (Figure 4a). The differences (Δ*I*) were calculated from the tabulated PREs (see section 4), with positive values (Δ*I* > 0) indicating higher PREs for TrpCM and negative values (Δ*I* < 0) indicating higher PREs for TyrCM. Even if it is unclear exactly where and how loop 11–12 samples various positions, the ΔI PREs unambiguously show changes in behavior. The most notable and largest positive PRE differences (Δ*I* > 0) are at the apparent entrance to the active site, namely V20 and V197, where PRE intensity ratios are ~2.5 fold larger in TrpCM (Figures 3b and 4a). PRE effects for TyrCM and TrpCM were also evaluated using a two time‐point rate calculation approach (Iwahara et al., 2006). While the time‐delay points were carefully optimized for CM to minimize excessive relaxation that could hinder data acquisition, PRE transverse methyl ^1^H magnetization rate (Γ_2_ = *R*_2,para_ − *R*_2,dia_) determination was not feasible due to practical limitations in measuring the methyl signals, given their wide range of relaxation rates. In the case of V197, based on other peaks that could be quantified, we estimated a lower limit of Γ₂ = 49 s^−1^ for TrpCM. For TyrCM, we were able to quantify most of the PRE rates, and V197 Γ₂ was determined to be 25 ± 6 s^*−*1^. Given that the V197 Γ₂ for TrpCM is approximately twice that of TyrCM, with comparable standard deviations, this highlights a pronounced difference in PRE effects between the two constructs in this region of the protein, confirming the Δ*I* observation from HMQC peaks (Figure 4a). Thus, relative to Tyr, binding of Trp activator biases loop 11–12 toward the active site surface, shown on the “underside” of the catalytic domain (Figure 4c). Other sites with positive Δ*I* values are I129, I31, and V211, with the latter two exhibiting bleached signals in TrpCM but only modest PREs in TyrCM (Figures 3c and 4a). By contrast, binding of Tyr leads to larger PREs (Δ*I* < 0) at a different region of the protein (Figure 4a). None of these differences are as large as for V20 and V197, but they form a cluster of residues that includes I103, V111, I237, I241, and V254, with I103 positioned slightly separated from the rest. As can be viewed in the data and mapped on the structure, this Tyr‐driven preference for loop 11–12 accessing this upper surface is modest, but it clearly drives the loop away from the lower surface, as seen in V20 and V197 (Figure 4c). Interestingly, these PRE differences are largely seen in the distal group of PRE sites (group 2) and suggest the Trp/Tyr differences in loop 11–12 positioning derive from its transient excursions from the “home position.”

We also considered whether these transient excursions of loop 11–12 could be explained with the classical allosteric models. We previously showed that TrpCM predominantly resides in the T‐state but dynamically switches to the R‐state at an equilibrium population of ~8% (Sapienza et al., 2023). Thus, a trivial explanation for the observed PRE differences between TrpCM and TyrCM, particularly Δ*I* > 0, would be that these changes arise from the R conformation, in which loop 11–12 adopts a different orientation (Figure 1c). To test this possibility, we measured TrpCM PREs in the absence of NaCl, which we have learned quenches excursions to the R‐state. If the R conformation is responsible for the PRE differences, then PREs of TrpCM at 0 mM NaCl should resemble those of TyrCM at normal buffer conditions and, in general, the PRE changes would be expected to scale with the R population. Our data show that TrpCM PREs do not vary proportionally with salt concentration. This is most evident with residue V197, which exhibits a large PRE increase relative to TyrCM (Figure 3): its PRE ratios are 5.2 ± 0.4 and 5 ± 0.2 in 150 and 0 mM NaCl, respectively. Similarly, residue I129 shows PREs of 2.0 ± 0.1 at high salt and 1.59 ± 0.04 in the absence of salt (see Figure S3 for further discussion). In addition, at pH 7.5, where we have previously reported a significant reduction in T‐to‐R switching (Sapienza et al., 2023), residues V197 and I129 exhibit PRE values of 5.9 ± 0.3 and 1.59 ± 0.06, respectively (Figure S4). These data indicate that the PRE‐detected movements occur from within the T state ensemble, and also support the use of 0 mM NaCl PREs in cases where PREs at 150 mM NaCl could not be determined (Table S1).

Taken together, the PREs show that loop 11–12 excursions depend on CM's effector‐bound status, with Trp allosterically promoting close approach of the loop to the lower surface where substrate entry likely occurs and Tyr allosterically redirecting the loop away from the active site. While TrpCM and TyrCM display highly similar overall conformational states despite their functional differences (Sapienza et al., 2023), the different PREs between TrpCM and TyrCM suggest that loop 11–12 adopts distinct conformational profiles depending on which effector is bound. The allosteric redirection of loop 11–12, which influences CM activity, is a striking example of purposeful modulation of a dynamic element by distal effector binding.

### 2.4 The allosteric loop and active site communicate through interprotomer interactions

In CM crystal structures in which parts of loop 11–12 are resolved, the positioning of the loop N‐ and C‐termini suggests that any potential communication between loop 11–12 and the active site likely occurs through intersubunit contacts (Figure 1). While the loop is in closer proximity to the active site in the presence of tryptophan (Figures 3c, 4a,c), the absence of a TrpCM wild‐type crystal structure presents challenges for determining whether these interactions arise from inter‐ or intra‐protomer contacts. To determine the nature of this interaction, we measured distances between residue S220, situated near the center of loop 11–12, and residue V197 near the active site in MD simulations of Trp‐ and Tyr‐bound CM (see section 4). Interestingly, TrpCM exhibited a subpopulation of intersubunit distance measurements between 5 and 15 Å, which was not observed in TyrCM measurements, showing excellent agreement with the PREs (Figure 4b). Specifically, 8% of all measured interprotomer distances between loop 11–12 and the active site in TrpCM fell within the 5–15 Å range, while less than 1% of such distances were observed for TyrCM, indicating that loop 11–12 moves much closer to the active site of the partner subunit when Trp is bound. Within these simulations, TrpCM predominantly remained in the T conformation, as the timescales were insufficient to overcome the kinetic barrier of the R conformation. Thus, the observed interprotomer contacts arose from interactions within the T‐state rather than the R‐state, consistent with our PRE interpretation. In contrast, all intraprotomer distances measured between loop 11–12 and V197 exceeded 25 Å for both Trp and Tyr‐bound forms (Figure 4b). The effective radius of the nitroxide spin label used in our PRE studies to induce line broadening in NMR signals is approximately 25 Å (Griffith & McConnell, 1966). The observation that all intraprotomer distances are greater than 25 Å suggests that intrasubunit interactions between loop 11–12 and the active site are unlikely. Instead, there is a high likelihood that PRE effects observed in regions near the active site arise from an interaction between loop 11–12 of one protomer and the vicinity of the active site of the opposite protomer, suggesting an intricate interplay between protomers that may be critical for CM's regulatory mechanism.

### 2.5 The allosteric loop alters the electrostatic environment of the active site

Loop 11–12's regulatory role appears to be attributed to intersubunit allosteric communication with regions near the active site (Figures 3 and 4), which is likely integral for CM function. It is remarkable how the removal of a charged residue (D215) in this flexible and unstructured loop suppresses the protein's activity in a pH‐reversible manner (Figure 2a), suggesting that loop 11–12 rearranges the protein's ionic environment to alter function. This led us to postulate that the loop excursions function to set up the active site to attract the densely negatively charged substrate. To test this hypothesis, we investigated the role of loop 11–12 in modulating TrpCM's charge distribution by analyzing the protein's surface electrostatic properties in solution.

**FIGURE 5:**
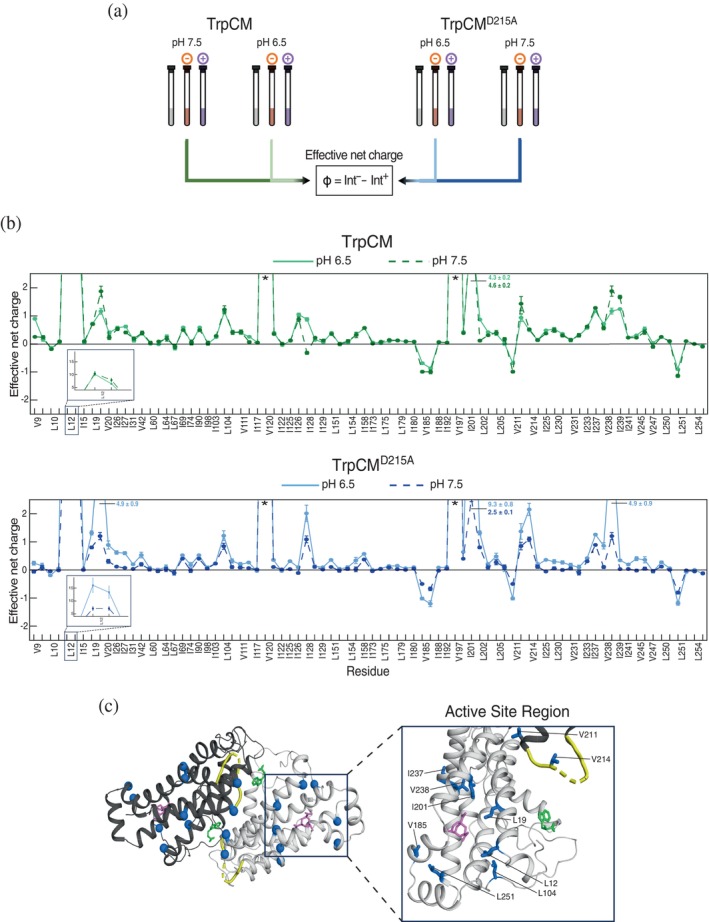
Loop 11–12 regulates CM's protein electrostatics. (a) Schematic of the sPRE experiment to obtain protein charge distribution. This experiment was conducted for TrpCM and TrpCM^D215A^ at pH 6.5 and pH 7.5. Three samples were prepared for each experiment type: sample without cosolute (diamagnetic, gray), sample mixed with a negatively charged cosolute (orange), and sample mixed with a positively charged cosolute (purple). (b) TrpCM (upper panel) and TrpCM^D215A^ (lower panel) effective net charges plotted against residues. Full lines represent data at pH 6.5, and dashed lines at pH 7.5. The zoomed‐in box highlights residue L12, which exhibited effective net charge values exceeding the plot limits. For other residues exceeding the plotting range, their corresponding effective net charge values are indicated numerically. Asterisks denote residues whose peak signals were bleached due to strong sPRE effects. (c) Residues exhibiting significant effective net charge differences in TrpCM^D215A^ relative to TrpCM are shown in blue. The left panel shows CM's homodimer, with the two subunits highlighted in different shades. The right panel provides a zoomed‐in view of the region where these residues are clustered. Most of the other subunit has been removed; only the loop from that subunit is shown. The criteria used to define significance are detailed in section 4. Substrate (magenta), loop 11–12 (yellow), and Trp (green) are highlighted for reference. PDB ID: 2CSM.

An innovative method was recently introduced for obtaining near‐surface electrostatic information on protein systems using NMR (Okuno et al., 2020; Yu et al., 2021). This method uses two solvent nitroxide spin labels—one negatively charged and the other positively charged—that can interact differentially with charged sites, providing insight into the electrostatic nature of the protein surface by producing PRE‐induced signal broadening. By monitoring these solvent PRE (sPRE) events, the protein's electrostatic distribution can be determined on a residue‐by‐residue basis. The calculation of effective near‐surface electrostatic potentials involves determining PRE rates for ^1^H transverse magnetizations (*R*_2_) using a two‐time‐point strategy (Iwahara et al., 2006; Okuno et al., 2020). As seen with the quantification of ^1^H methyl PRE rates, this pure *R*₂ approach proved impractical in our study due to substantial signal broadening and peak disappearance caused by the wide range of methyl relaxation rates, along with the increased number of samples. Therefore, we adopted a modified strategy involving the collection of ^1^H‐^13^C HMQC spectra of paramagnetic and diamagnetic samples to minimize peak disappearance and enable rapid acquisition for different CM forms (Figure 5a). As a proxy for calculating electrostatic potentials, we employed a simple measure of surface charge directly from ^1^H‐^13^C HMQC peak intensities: $\phi =I_{\text{ratio}}^{-}-I_{\text{ratio}}^{+}$, where $I_{\text{ratio}}^{-}$ and $I_{\text{ratio}}^{+}$ represent the intensity ratios derived from the sPRE effect produced by the negatively and positively charged cosolutes, respectively, integrated into a unified parameter, ϕ, that reports on CM's surface effective charge distribution. A positive final value (ϕ>0) indicates a net positively charged region, while a negative value (ϕ<0) indicates a net negatively charged region. This approach was specifically implemented to study tryptophan‐bound forms of CM, namely the wild‐type and D215A forms, as this is where we observe pronounced pH‐dependent changes in enzymatic activity (Figure 2a). Since the D215A mutation exerts an inhibitory effect on the otherwise active TrpCM at pH 7.5 but not at pH 6.5, we sought to investigate changes in electrostatics distribution that could explain TrpCM activity changes. After comparing electrostatics parameters for TrpCM^D215A^ and TrpCM at pH 6.5 and pH 7.5, we found significant discrepancies between CM forms with different activity profiles.

TrpCM, which displays an active and hyperbolic activity profile at both pH 6.5 and pH 7.5 (Figure 2a), showed a largely unchanged consistent pattern in effective net charge distribution at both pHs (Figure 5b), suggesting that the wild‐type enzyme maintains a stable electrostatic environment to support its function at the two pH values. It should be noted that the apparent *K*_m_ of TrpCM differs across the pH values (Figure 2a). It is unclear exactly what underlies the difference in *K*_m_ values, but it appears not to be from the surface charge properties as captured by the methyl‐detected sPREs. In contrast, introducing the D215A mutation within loop 11–12 leads to marked differences in effective net charge distribution across CM at pH 6.5 and 7.5 (Figure 5b) that mirror the observed loss of activity upon raising the pH. Notably, many of the residues showing the most pronounced changes—L12, L19, L104, V185, I201, V211, V214, I237, V238, and L251—are located in the vicinity of the active site (Figure 5c). At pH 6.5, residues L12, L19, L104, I201, V214, I237, and V238 exhibit significantly higher positive effective net charge compared to pH 7.5. Interestingly, many of these residues fall within or near the region sampled by loop 11–12 in the presence of the activator Trp, defined above as group 2. This spatial correlation suggests a potential coupling mechanism in which loop 11–12 approaches regions near the active site to facilitate a favorable electrostatic environment, thereby promoting accessibility of the negatively charged substrate and enabling proper protein function.

It is important to consider that, given the charged nature of the sPRE probes, they could preferentially bind to highly charged pockets of the protein in addition to interacting with surfaces, which could potentially bias the observed differences in effective net charge. Subtle peak shifting in residues V111 and V245 suggests that such binding events, or another perturbation causing these shifts, might occur across all wild‐type and mutant samples (Figure S6). Additionally, V111 and V245 signals are far from bleached, which would be expected for significant binding that might contaminate PREs at other sites. Therefore, it is unlikely that a binding event is responsible for the main pattern of observed PREs.

To further support our sPRE electrostatics analysis, we collected a series of amide backbone NMR spectra for both TrpCM and TrpCM^D215A^ across a pH range of 6.0–8.0. This experiment allowed the identification of specific residues throughout the protein that exhibit differential responses to pH‐dependent titration. Several residues exhibited significantly altered behavior in TrpCM^D215A^ compared to the wild type (Figures S7 and S8). One such residue is V197, which is one of the probes approached by loop 11–12 in TrpCM, as evidenced by large PRE effects (Figure 3b,c). Additionally, residues K190, E203, and E210, located within or near regions showing pronounced changes in effective net charge in TrpCM^D215A^, also displayed significant differences in their titration curves relative to TrpCM (Figure S8). While the subtle shift differences observed do not implicate specifically which side chains are deprotonated, these observations provide additional evidence that loop 11–12 allosterically modulates the electrostatic landscape of CM, which is likely important for maintaining optimal enzyme activity.

In TrpCM, D215 remains predominantly distant from the active site. Therefore, its influence on activity is allosteric, and unlike many observations of allostery, here it is possible to pinpoint the specific protein element producing the allosteric effect. Furthermore, it appears that there is an electrostatic component to the allosteric mechanism, which likely combines structural repositioning, dynamics, and either direct electrostatic perturbation or an indirect, extensive rearrangement of an electrostatic network in CM's catalytic domain that relies on charge at position 215 of loop 11–12. Combined with the flexibility of loop 11–12 and the role of D215 in modulating enzymatic activity, these findings point to a sophisticated allosteric mechanism evolved by CM, which may offer insights into how allostery operates in classical allosteric systems, providing a potential framework for understanding and exploring allosteric regulation in other proteins.

## 3 DISCUSSION

CM's loop 11–12 behavior demonstrates that allosteric mechanisms beyond traditional structural rearrangements can play critical roles in regulating protein activity. Braus and colleagues previously highlighted the unexpected yet essential role of loop 11–12 in CM function (Helmstaedt et al., 2002). In the present study, we provide further insights on loop 11–12's functional role and identify an allosteric mechanism by which it communicates with key regulatory regions of the protein to influence enzymatic activity. Using PRE NMR, we showed that the loop's spatial orientation is influenced by effectors bound more than 20 Å away from the loop termini (Figure 3). In the presence of the activator Trp, the loop exhibits excursions toward the active site that are absent in both apoCM and TyrCM. We previously showed that active site residues V197 and V20, which exhibit high PREs in TrpCM (Figure 3), also display deviations from the main T‐R switching residues, suggesting that their relaxation properties are influenced by an alternative dynamic mechanism (Sapienza et al., 2023). It is highly likely that the proximity of loop 11–12 to the active site modulates the dynamics of these and surrounding residues to facilitate substrate access. The distinctive motion of loop 11–12 observed in TrpCM aligns with prior work by Boehr and colleagues, who reported lower methyl‐axis order parameters for a residue within loop 11–12, T217, in TrpCM, indicating greater flexibility relative to apoCM and TyrCM (Gorman et al., 2020). Notably, PRE effects across different regions of the protein (groups 1 and 2 above) support the idea that the loop samples different ensembles of conformations rather than executing a binary “open‐closed” hinge motion. Thus, loop 11–12 does not act as a locking mechanism upon activator binding, but rather explores a broad conformational landscape, highlighting the complexity of loop‐assisted allosteric regulation.

Further insights into the behavior of loop 11–12 emerged from MD simulations, which revealed that its communication with the active site could occur via interprotomer contacts (Figure 4b). These findings are consistent with the PRE data, which indicate that loop 11–12 makes intermittent close approaches to several residues within the active site. This manner of intersubunit communication between the distal loop and active site provides insight into protein cooperativity mechanisms, a hallmark of CM's allosteric behavior. Multiple studies show that CM exhibits interprotomer cooperativity, particularly in its apo and Tyr‐bound forms (Graf et al., 1995; Helmstaedt et al., 2002; Sapienza et al., 2021; Schmidheini et al., 1990; Schnappauf et al., 1997; Schnappauf, Lipscomb, & Braus, 1998). Herein, we have shown that introducing the D215A mutation within loop 11–12 induces an inhibitory effect partially compensated by a cooperative mechanism, as evidenced by a sigmoidal‐shaped activity curve (Figure 2a). It is, therefore, plausible that loop 11–12 also contributes to the control of allosteric cooperativity in CM. This could be further validated by employing the mixed‐labeled dimer (MLD) strategy recently developed by our group, which involves tethering an NMR‐detectable CM monomer to an NMR‐silent partner via click chemistry (Sapienza et al., 2021). The MLD approach can be leveraged to explore loop 11–12 intersubunit accessibility through PRE measurements across subunits and by analyzing scenarios where substrate binding occurs on only one subunit of the homodimer, thereby allowing investigation of cooperativity coordinated by loop 11–12 and other regions of the protein.

Our findings reveal that the loop not only dynamically accesses the active site and other regions of the protein but that this motion likely serves a specific functional role. It is remarkable how TrpCM^D215A^ exhibits an inhibitory effect at pH 7.5, yet its activity is restored to wild‐type levels at pH 6.5 (Figure 2a). Given that this pH‐dependent reversibility arises from mutating a charged residue within loop 11–12, this change may alter the protein's electrostatic properties and modulate enzymatic function. Using a modified sPRE NMR strategy to investigate the electrostatic surface of CM, we demonstrate that loop 11–12 rewires the protein's electrostatic landscape, an effect that is especially pronounced in TrpCM^D215A^. At pH 6.5, where TrpCM^D215A^ exhibits a wild‐type‐like activity profile, a marked increase in positive effective net charge is observed relative to wild type, particularly in regions around the active site and loop 11–12 (Figure 5). With the limited amount of electrostatic and protonation information collected here, it is not fully clear how a D215A‐linked activity enhancement occurs at pH 6.5. However, we can propose a simple scheme that incorporates the observed overall increase in positive charge in regions near the active site that only occurs in TrpCM^D215A^. We propose that the increased charge, or “positive cloud” at pH 6.5, boosts access of the negatively charged substrate to the active site, rescuing activity from a mutation that otherwise loses a side‐chain carboxylate that presumably enters into an electrostatic network to promote activity. At pH 7.5, however, reduced availability of free protons dissipates the “cloud” and eliminates the rescue of function. In the case of the wild‐type enzyme, the electrostatics distribution remained largely consistent across residues at pH 6.5 and 7.5, even though the apparent *K*_m_ of TrpCM varied at different pHs. It is fascinating how the presence of D215 eliminates charge accumulation at pH 6.5, as if the carboxylate at D215 engages the network in a robust fashion to prevent certain protonations at pH 6.5. The change in *K*_m_ may have arisen from other mechanistic factors involved in CM catalysis that are not fully captured by our methyl‐based sPRE approach. Nevertheless, our PRE and sPRE data strongly suggest that loop 11–12 contributes, at least in part, both structurally and functionally to the allosteric regulation of CM. Further investigations will be necessary to uncover additional processes that may support this mechanism.

It is unexpected that a distal, flexible loop can respond to effectors and regulate enzyme activity. Loop 11–12 is essentially “external” to the core fold of the protein since the bulk of the loop is solvent‐exposed and does not pack. Regulation by this external element, thus, stands in contrast with classical mechanisms of allostery, in which conformational changes are typically integrated into structured, packed, and more interior regions of the protein. Even modern illustrations of MWC and KNF models, for example, reflect structure‐wide shape changes. These observations, therefore, raise a broader question: is allostery in CM, and potentially in other proteins, governed primarily by internal structural rearrangements, external elements, or a combination of both? Classical models of allostery typically emphasize internal conformational changes as the key drivers of long‐range communication. However, our data suggest that external contributors, such as flexible regions like loop 11–12, may also play critical roles in mediating allosteric signaling. A synergistic model, incorporating both internal and external factors, may be necessary to fully understand communication between distal sites within some proteins. We argue that flexible loops, often overlooked in structural studies, merit greater attention, as they may conceal valuable regulatory features contributing to protein function, such as found here for yeast chorismate mutase. Integrating external contributors into models of allosteric regulation may not only deepen our understanding of known allosteric systems but also reveal latent allosteric behavior in proteins not previously characterized as such, supporting the conception that all proteins may possess the inherent capacity for allosteric regulation (Gunasekaran et al., 2004).

**FIGURE 6:**
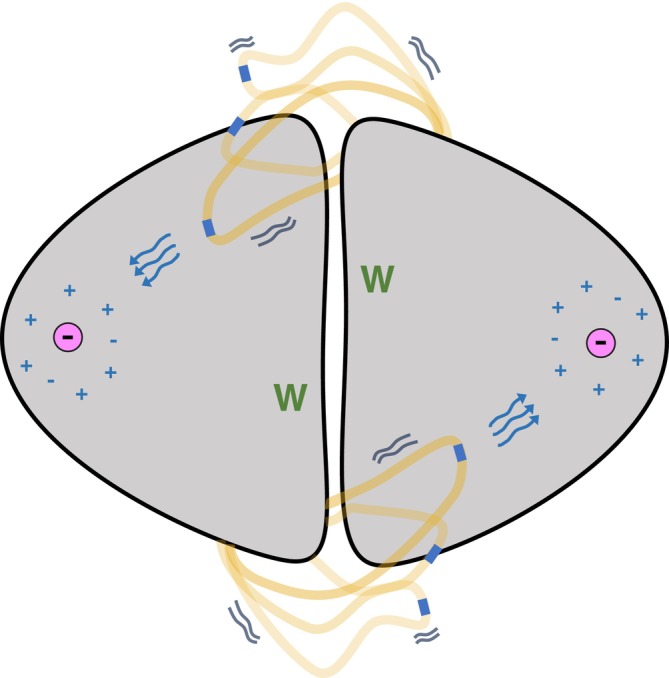
Proposed TrpCM allosteric model. Loop 11–12 communicates with regions in the active site through interprotomer contact. The blue region in the loop represents D215 and its role in maintaining the proper electrostatic balance of the active site. This effect is regulated by the binding of the effector tryptophan (green), which binds far from both the loop and the active site. This creates an allosteric communication mechanism between three critical hotspots of the protein: the effector binding site, loop 11–12, and the active site.

In summary, we propose a model wherein a flexible allosteric loop undergoes effector‐dependent conformational sampling to reorganize electrostatic landscapes and assist in protein activity regulation (Figure 6). This non‐classical mode of allostery—dominated by the dynamic sampling of a flexible loop as opposed to conformational change—not only broadens our understanding of allosteric regulation but also points to the possibility of future design principles for engineering allosteric systems. By emphasizing the role of local electrostatics and dynamic flexibility, this work may inform future efforts in rational drug and protein design.

## 4 MATERIALS AND METHODS

### 4.1 Expression and purification of CM

BL21*(DE3) cells were transformed with pET21a plasmids containing a TEV‐cleavable CM gene. Protein expression followed the M9+ protocol under a deuterated background and isoleucine, leucine, and valine (ILV) methyl isotopic enrichment, as previously outlined (Cai et al., 2016; Sapienza et al., 2023). Purification was performed using affinity and size‐exclusion chromatography, as described previously (Sapienza et al., 2021). Point mutations were generated using PCR site‐directed mutagenesis and expressed and purified as described above.

### 4.2 CM activity assays

Enzymatic activity assays were conducted in triplicates for all CM variants, including mutant forms, under NMR Buffer conditions (25 mM NaHPO_4_, 150 mM NaCl, 1 mM EDTA, 2.8 mM NaN_3_, 1 mM DTT, and 99.9% H_2_O) at pH 6.5 or 7.5 and 22 °C, adhering to the previously established protocols (Sapienza et al., 2021). CM^S220C‐TEMPO^ was tested for activity before preparing and conducting the PRE experiments (Figure S5 and Table S2).

### 4.3 PRE NMR‐sample preparation and analysis

To introduce a paramagnetic probe into loop 11–12 for spatial mapping, an 80 mM stock of the nitroxide spin label in acetonitrile, 4‐maleimido‐TEMPO (Toronto Research Chemicals), was prepared. A solution containing 1 mL of 10 mM Tris–HCl buffer, pH 7.5 (labeling buffer) was prepared with 4‐maleimido‐TEMPO at 15‐fold the final protein concentration in the reaction and mixed in a foil‐covered conical tube. Attachment of 4‐maleimido‐TEMPO on CM's loop 11–12 was achieved by adding 500 μL of CM^S220C^ into a G25 column (Cytiva) and eluting the protein with 2 mL of labeling buffer collected into the foiled conical tube with ~1 mL labeling buffer and 4‐maleimido‐TEMPO, for a total sample volume of ~3 mL and a final protein concentration of 30–50 μM. The labeling reaction was left shaking at room temperature for 30 min at 90 rpm. Afterward, the protein was buffer‐exchanged by adding 30 mL NMR buffer (25 mM NaHPO_4_, 150 mM NaCl, 1 mM EDTA, 2.8 mM NaN_3_, 99.9% D_2_O, and pH 6.5) in a stepwise fashion into a 10 kDa Amicon filter (Millipore Sigma) and spinning at 3500 rpm, ensuring the removal of any unreacted 4‐maleimido‐TEMPO. Modifications to NMR buffer conditions to enhance methyl signal visibility in some samples are detailed in Figure S3. When required, tryptophan and tyrosine were added to a final concentration of ~2 mM into separate CM samples. Protein samples were placed into 3 mm NMR tubes (Norell) to a final concentration of ~200 μM.

After NMR data acquisition for the paramagnetic sample, sodium ascorbate was added to a final concentration of 2 mM to generate a diamagnetic form proceeding at room temperature for 3 h without mixing. Afterward, the diamagnetic portion of the NMR PRE experiment was collected.

NMRPipe (Delaglio et al., 1995) and NMRView (Johnson & Blevins, 1994) were used for data processing and analysis. PRE profiles were obtained by determining the intensity ratio ($I_{\text{ratio}}$) of individual residues using in‐house developed Python scripts employing the following equation:

(1) $$I_{\text{ratio}}=\frac{I_{\mathrm{dia}}}{I_{\text{para}}}*c,$$

where $I_{\mathrm{dia}}$ and $I_{\text{para}}$ represent the peak intensity of individual residues in the diamagnetic and paramagnetic spectra, respectively. The variable c corrects for variations in protein concentration across samples by calculating the ratio of the protein concentration in the paramagnetic sample to that in the diamagnetic sample. Uncertainty in intensity ratios was calculated as follows:

(2) $$\sigma_{I_{\text{ratio}}}=\sqrt{{\left(\frac{\sigma_{I_{\text{para}}}}{I_{\text{para}}}\right)}^{2}+{\left(\frac{\sigma_{I_{\mathrm{dia}}}\cdot I_{\mathrm{dia}}}{I_{\text{para}}}\right)}^{2},}$$

where $\sigma_{I_{\text{para}}}$ and $\sigma_{I_{\mathrm{dia}}}$ are the uncertainties in paramagnetic and diamagnetic spectra, respectively, determined from baseplane noise. Results were plotted using MATLAB software (MathWorks).

### 4.4 sPRE‐NMR sample preparation and electrostatics analysis

~200 μM protein samples of TrpCM and TrpCM^D215A^ in NMR buffer (25 mM NaHPO_4_, 150 mM NaCl, 1 mM EDTA, 2.8 mM NaN_3_, and 99.9% D_2_O) at either pH 6.5 or pH 7.5 were prepared. For each CM form, three sample types were prepared: one containing 5 mM 4‐amino‐TEMPO (positively charged cosolute, Sigma Aldrich), another with 5 mM 4‐carboxy‐TEMPO (negatively charged cosolute, Sigma Aldrich), and one that was cosolute‐free (diamagnetic). The concentration of each cosolute stock solution was determined prior to adding it to the final protein sample using the PULCON method (Wider & Dreier, 2006). To determine the influence of each cosolute on peak broadening and thus define the electrostatic environment of each CM variant, peak intensities for each NMR spectrum were measured, and the sPRE effects were calculated using Equation (1). The contributions from both positively and negatively charged cosolutes were combined using the following expression:

(3) $$\phi =I_{\text{ratio}}^{+}-I_{\text{ratio}}^{-},$$

where $I_{\text{ratio}}^{+}$ and $I_{\text{ratio}}^{-}$ are the sPRE contributions of the positively and negatively charged cosolutes, respectively. To determine the uncertainty in intensity ratios, Equation (2) was applied initially for each calculated intensity ratio $I_{\text{ratio}}^{+}$ and $I_{\text{ratio}}^{-}$, and errors were propagated by

(4) $$\sigma_{\phi }=\sqrt{{\left(\sigma_{I_{\text{ratio}}^{+}}\right)}^{2}+{\left(\sigma_{I_{\text{ratio}}^{-}}\right)}^{2},}$$

where $\sigma_{I_{\text{ratio}}^{+}}$ and $\sigma_{I_{\text{ratio}}^{-}}$ represent the uncertainties for sPRE contributions of the positively and negatively charged cosolutes, respectively.

To identify residues exhibiting significant differences in effective net charge (ϕ) between TrpCM^D215A^ and TrpCM, we applied the following multi‐step criterion. First, to assess how the pH‐dependent behavior of the mutant differed from that of the wild‐type form, we calculated the effective net charge difference per CM form using the equation:

(5) $$∆\phi =\phi_{\mathrm{pH}\;6.5}-\phi_{\mathrm{pH}\;7.5},$$

where $\phi_{\mathrm{pH}\;6.5}$ and $\phi_{\mathrm{pH}\;7.5}$ are the effective net charges at pH 6.5 and 7.5, respectively. Next, we obtained the absolute difference of differences to quantify how the pH response varied between TrpCM^D215A^ and TrpCM:

(6) $$∆∆\phi =∆\phi_{{\text{TrpCM}}^{D215A}}-∆\phi_{\text{TrpCM}}.$$

Residues with ∆∆ϕ≥0.4 were considered to show significant divergence between TrpCM^D215A^ and TrpCM. As a final filter, only residues for which all four samples had standard deviations ≤20% of their respective effective net charge values were retained. Residues that met all criteria were designated as showing significantly different effective net charge between the two CM forms and were highlighted in blue in the crystal structure shown in Figure 5c.

### 4.5 NMR spectroscopy experiments

All NMR spectra were recorded on a Bruker Avance III HD 850 MHz spectrometer equipped with a TCI H‐C/N‐D 5 mm cryoprobe, and data were controlled via TopSpin 3.5 pl 7. ILV ^1^H‐^13^C HMQC spectra were acquired at 15°C following the pulse sequence described in prior literature (Kay, 2019). Chemical shift perturbations (CSPs) presented in Figure 2c were calculated as previously described with adjustments for ^13^C chemical shifts using a scaling factor of 0.133 (Mulder et al., 1999). ^1^H CPMG experiments were acquired at 15°C, processed, and analyzed as previously described (Sapienza et al., 2023).

### 4.6 MD simulations and distance measurements

The crystal structure analyzed was obtained from the Protein Data Bank (PDB ID: 2CSM). Structure reconstruction, ligand addition, and MD simulation details have been published previously by us (Sapienza et al., 2023). In brief, MD simulations were conducted with the GROMACS 2020.3 software package (Hess et al., 2008; Pronk et al., 2013). The proteins were parameterized using the CHARMM36 force field (Best et al., 2012), solvated in the SPCE water model, and neutralized with sodium and chloride ions. Long‐range electrostatics were treated with the particle mesh Ewald (PME) method, applying a 10 Å cutoff for non‐bonded interactions. The system first underwent equilibration under constant volume and temperature (NVT), followed by constant pressure and temperature (NPT) conditions at 300 K and 1 atm. Each system was simulated in triplicate for at least 350 ns with a 2 fs integration timestep, discarding the first 50 ns to ensure proper equilibration. Trajectories were saved every 1 ns for subsequent analyses. To evaluate the distance between loop 11–12 and the active site for Trp‐ and TyrCM, we determined the coordinates of the S220 hydroxyl group O^Ɣ^ proton (loop 11–12) and the V197 methyl group C^Ɣ1^ proton (active site) across over 600 snapshots of Trp‐ and TyrCM for all sets of inter‐ and intramolecular pairs: loop 11–12 chain A vs. active site chain A/B and loop 11–12 chain B vs. active site chain A/B. These coordinates were used to calculate the Euclidean distances between loop 11–12 and the active site. Distances were categorized into inter‐ and intraprotomer measurements and plotted using MATLAB. This analysis was performed using in‐house developed Python and MATLAB scripts, available upon request.

## AUTHOR CONTRIBUTIONS

**Darex J. Vera‐Rodríguez:** Conceptualization; investigation; writing – original draft; methodology; validation; visualization; software; formal analysis; project administration; data curation. **Paul J. Sapienza:** Investigation; writing – review and editing; data curation; conceptualization. **Konstantin I. Popov:** Investigation; writing – review and editing; data curation. **Andrew L. Lee:** Conceptualization; funding acquisition; writing – review and editing; validation; visualization; project administration; supervision; resources.

## CONFLICT OF INTEREST STATEMENT

The authors declare no conflicts of interest.

## Supporting information


**Data S1.** The supplementary material file (VeraRodriguez_AllostericLoop_SI_Review.pdf) includes figures and tables presenting additional results that support the findings described in the main text. These encompass NMR spectra, processed NMR data displayed in figures and tables, and enzymatic activity assays.

## Data Availability

The data supporting the findings of this study are included within the manuscript. The scripts used for data processing and analysis are available upon request.
