# Supplementary material for: A flexible, allosteric loop regulates protein activity and rewires electrostatics
Source: Protein Sci. 2025 Sep 24;34(10):e70315. doi: 10.1002/pro.70315 (PMC12459221; doi:10.1002/pro.70315)
Supplement: Supplementary file 1 — Data S1. The supplementary material file (VeraRodriguez_AllostericLoop_SI_Review.pdf) includes figures and tables presenting additional results that support the findings described in the main text. These encompass NMR spectra, processed NMR data displayed in figures and tables, and enzymatic activity assays. [file PRO-34-e70315-s001.pdf]

SUPPLEMENTAL INFORMATION

A flexible, allosteric loop regulates protein activity and rewires electrostatics

Darex J. Vera-Rodríguez[1], Paul J. Sapienza[2], Konstantin I. Popov[1,2], and Andrew L. Lee[1,2,*]

[1]Department of Biochemistry and Biophysics, University of North Carolina at Chapel Hill, Chapel Hill, NC 27599, United States

[2]Division of Chemical Biology and Medicinal Chemistry, University of North Carolina at Chapel Hill, Chapel Hill, NC 27599, United States

[*]Corresponding Author

Andrew L. Lee

4109 Marsico Hall, CB #7363, Chapel Hill, NC, 27599-7363

(919) 966-7821

drewlee@unc.edu

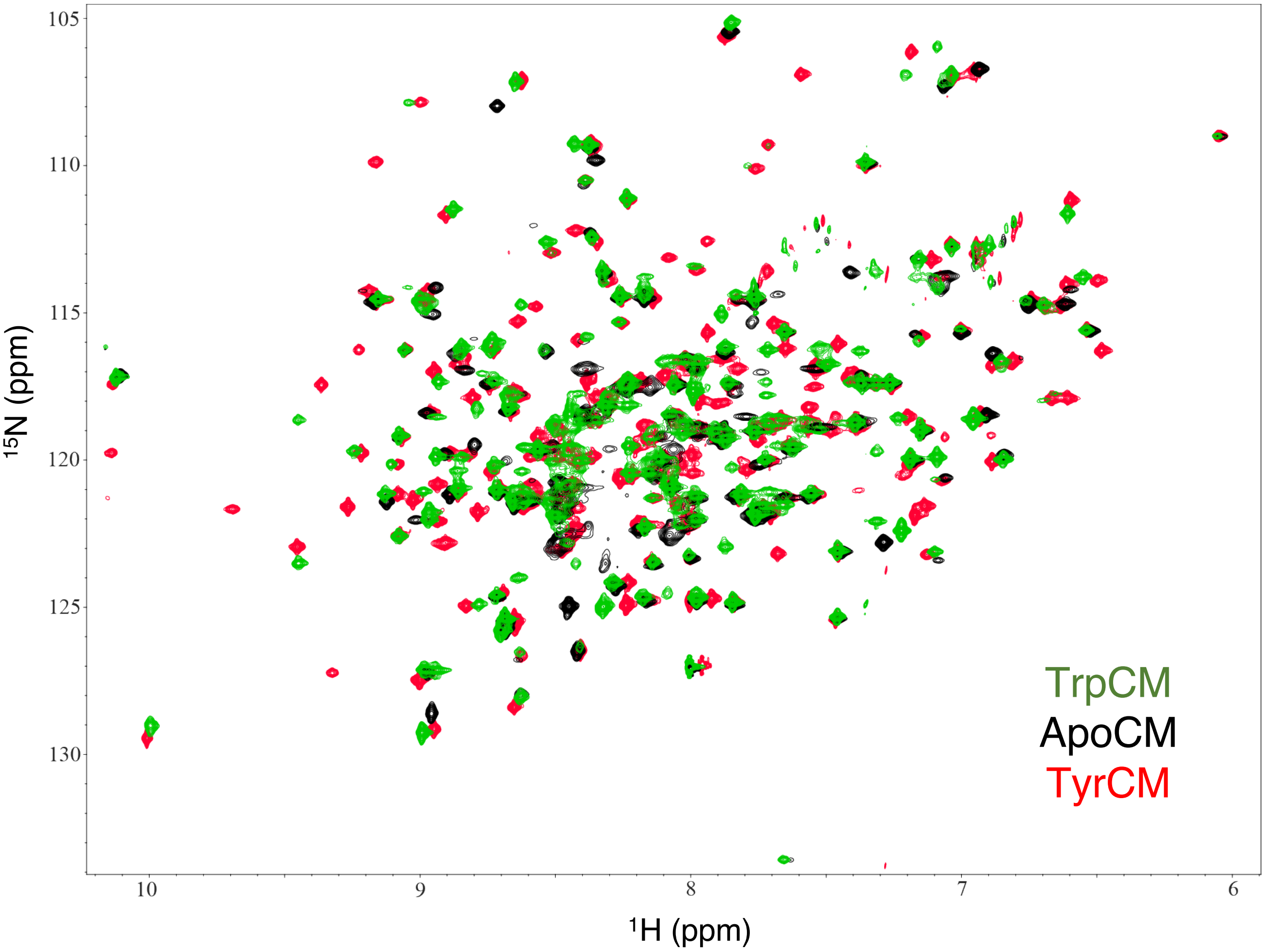

Figure S1. Missing TrpCM and apoCM amide signals correspond primarily to loop 11-12 and the effector binding region (EBR). ^{1}H-^{15}N HSQC spectra are shown for TrpCM (green), apoCM (black), and TyrCM (red). TyrCM displays a greater number of peaks compared to TrpCM and apoCM, indicating that the apo and Trp-bound forms of CM experience greater protein dynamics than TyrCM. We previously reported challenges in assigning several TrpCM amide resonances due to signal disappearance, likely caused by high flexibility within certain regions of the protein (Sapienza et al. 2021). These disappearing peaks were predominantly located within loop 11-12 and the EBR, suggesting that these regions exhibit significant structural flexibility. We further validated these observations on protein dynamics using methyl probes (Sapienza et al. 2023).

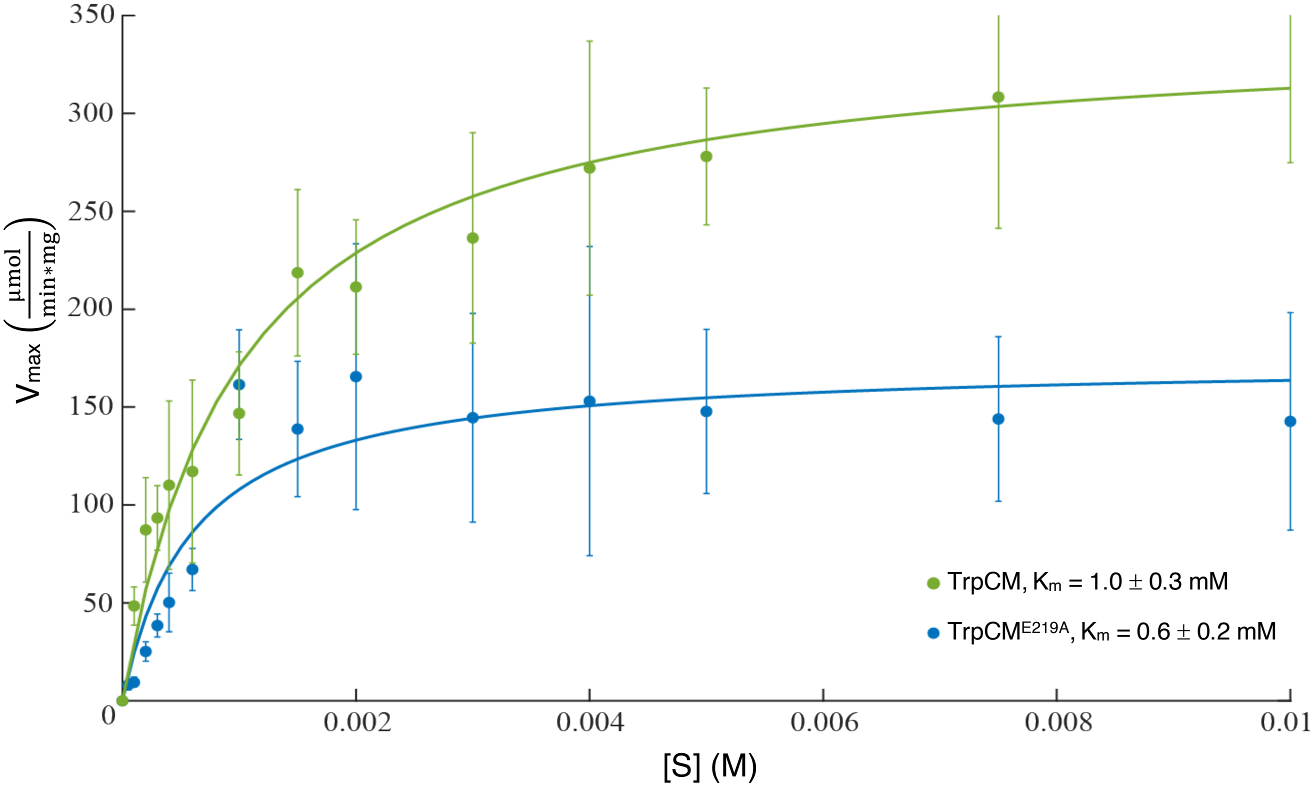

Figure S2. TrpCM and TrpCM^{E219A} activity assays at pH 7.5. Activity measurements were performed as described in the main text under the following buffer conditions: 25 mM $NaHPO_4$, 150 mM NaCl, 1 mM EDTA, 2.8 mM NaN_3, 1 mM DTT, pH 7.5, and 22°C. The K_m values are nearly identical, indicating that E219A does not impact substrate binding.

Figure S3 Supporting Discussion: Collection of TrpCM$^{S220C\text{-}TEMPO}$ methyl spectra under standard buffer conditions (including 150 mM NaCl; see Figure S3) resulted in multiple missing peaks due to signal broadening caused by TEMPO. The missing signals correspond to residues previously identified as undergoing global T-R switching in TrpCM (Sapienza et al. 2023). Thus, introduction of the PRE probe within loop 11-12 amplifies this dynamic behavior, leading to the observed peak loss. To address this, we reduced the NaCl concentration to near 0 mM, which successfully recovered the missing peaks by attenuating the T-to-R switching. The PRE data presented in Figure 3C of the main text includes a combination of measurements acquired at both 150 mM and 0 mM NaCl, as detailed in Table S1.

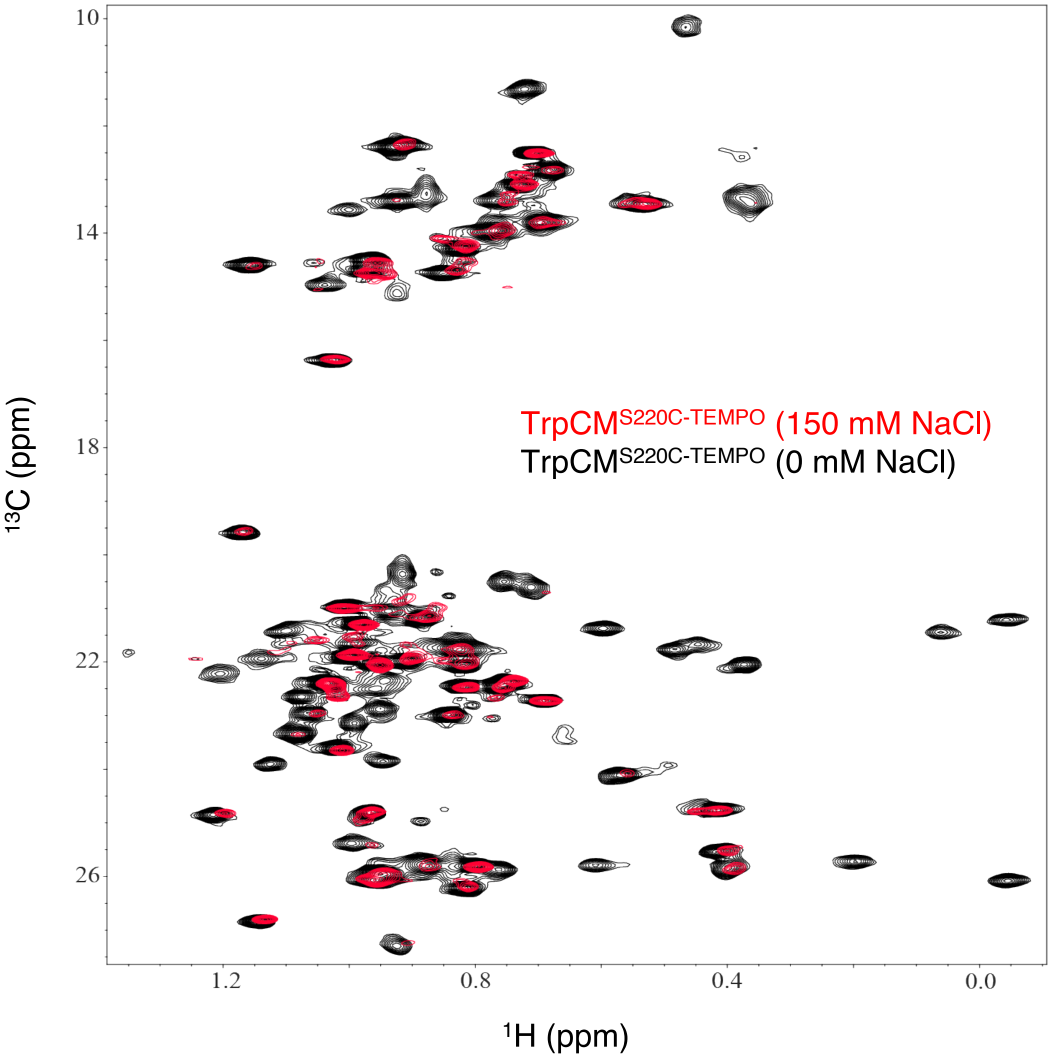

Figure S3. ILV ^1H-^{13}C HMQC overlay of TrpCM$^{S220C\text{-}TEMPO}$ at high and low NaCl concentrations shows missing peaks at high NaCl concentration. Methyl NMR spectra of TrpCM$^{S220C\text{-}TEMPO}$ in its diamagnetic form was acquired at high (red) and low (black) NaCl concentrations. Buffer conditions were 25 mM $NaHPO_4$, 1 mM EDTA, 2.8 mM NaN_3, pH 6.5, and 99.9% D_2O with or without NaCl as indicated. Notably, high salt concentrations caused multiple peaks across the spectrum to disappear. These peaks were visible at low salt concentrations, enabling PRE analysis. No significant changes in activity were detected between these two conditions (Figure S5 and Table S2). TEMPO attachment to S220C and sample preparation were performed as described in the main text.

Table S1. TrpCM residues for which PRE intensity ratios were calculated using 150mM NaCl (red) and 0mM NaCl (black) and plotted in Figure 3C in the main text.

Residue	
9CG2	175CD1
10CD2	175CD2
12CD1	179CD1
12CD2	180CD1
15CD1	185CG2
20CG1	188CD1
27CD1	197CG1
31CD1	197CG2
42CG1	201CD1
60CD1	202CD2
60CD2	205CD1
64CD1	205CD2
64CD2	211CG2
74CD1	214CG2
98CD1	225CD1
103CD1	230CD1
104CD1	230CD2
104CD2	231CG1
111CG1	231CG2
111CG2	237CD1
120CG1	238CG1
128CD1	241CD1
128CD2	245CG1
129CD1	245CG2
151CD2	247CG2
154CD1	250CD1
154CD2	251CD1
158CD1	254CD1
173CD1	254CD2

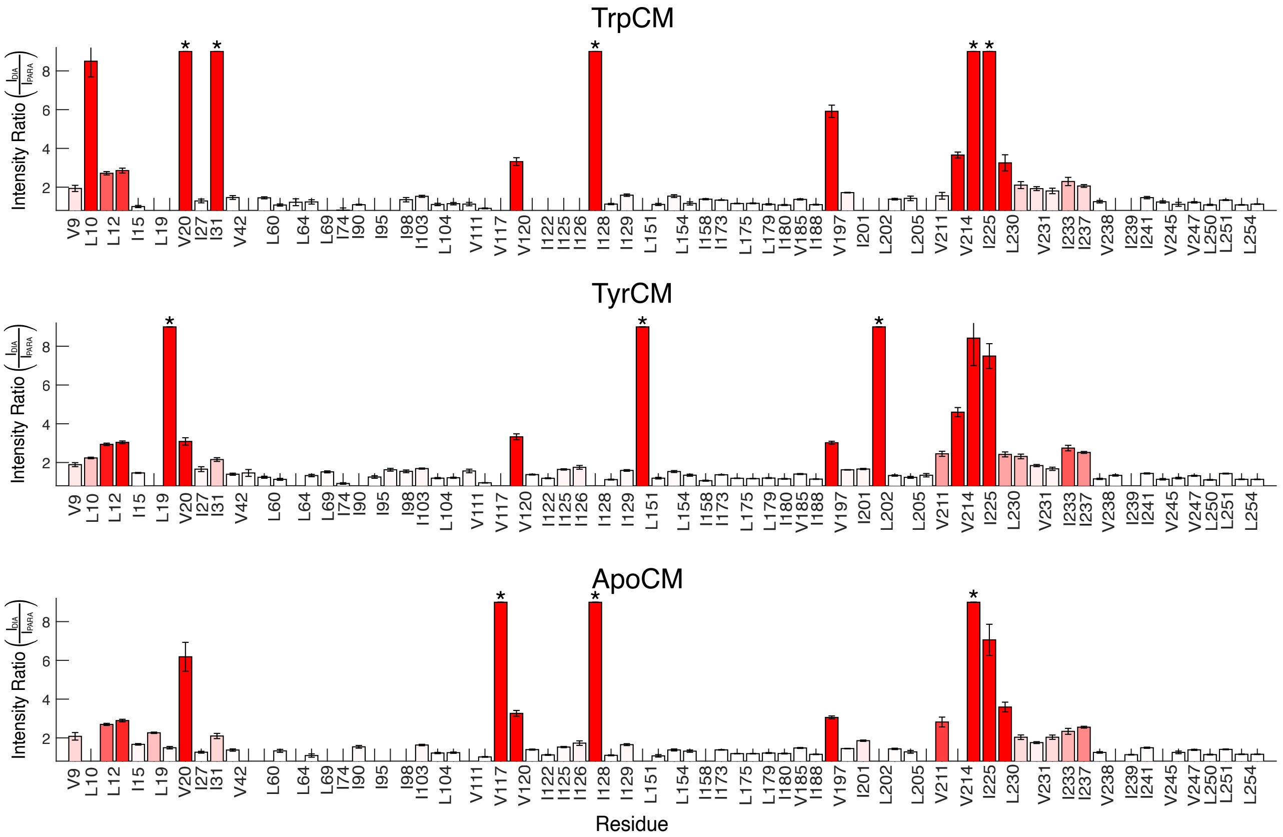

Figure S4. Loop 11-12 exhibits similar behavior at pH 7.5 as observed at pH 6.5. PRE profiles for CM methyl residues are shown for TrpCM (upper panel), TyrCM (middle panel), and apoCM (bottom panel) at pH 7.5. Buffer conditions were 150mM NaCl, 25 mM $NaHPO_4$, 1 mM EDTA, 2.8 mM NaN_3, and 99.9% D_2O. Residues V20, V31, and V197 display higher PRE values in TrpCM compared to TyrCM and apoCM, consistent with our findings at pH 6.5 (Figure 3C in the main text). Bars are color-coded using a red gradient to highlight variations in PRE intensity. Residues marked with an asterisk indicate complete signal loss due to strong PRE effects. Empty spaces represent residues for which NMR assignments were unavailable in the respective CM forms.

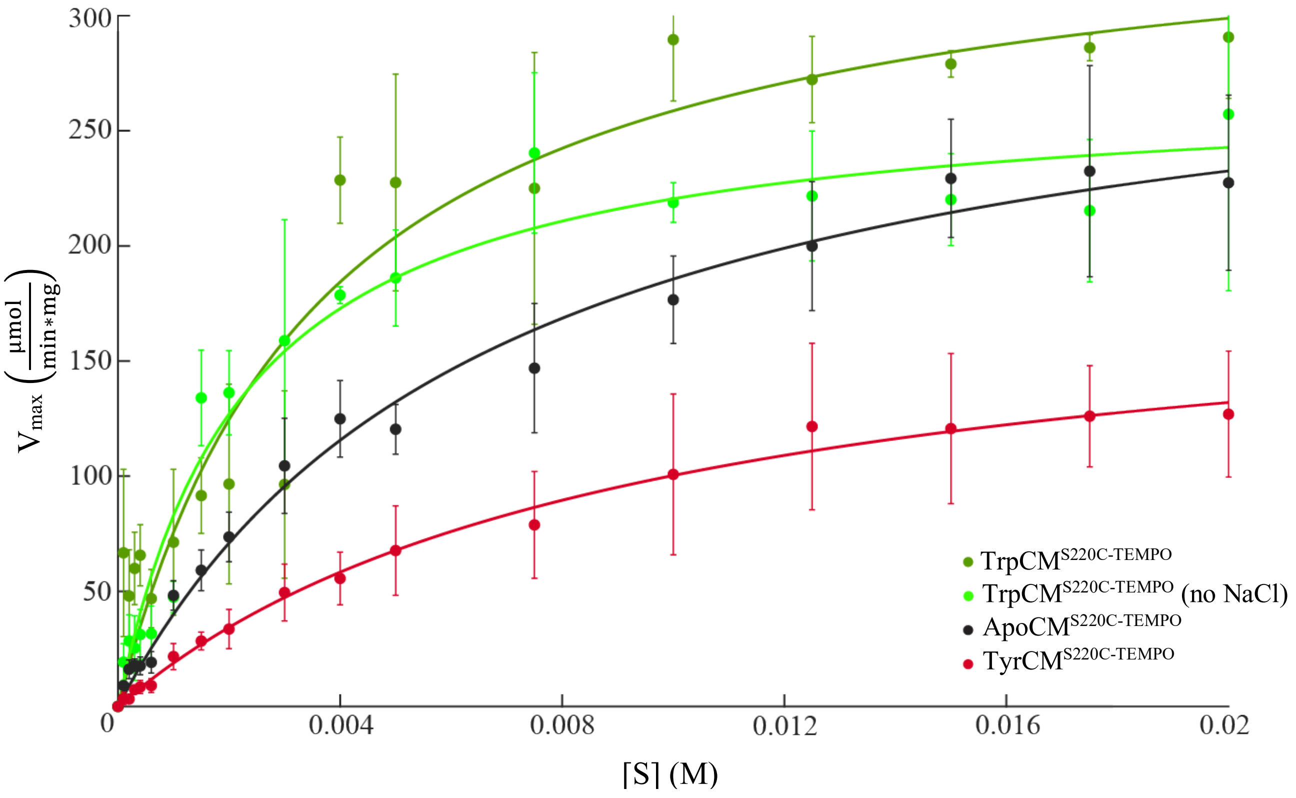

Figure S5. $CM^{S220C\text{-}TEMPO}$ Activity Assays. Attachment of TEMPO to CM^{S220C}, to mimic NMR conditions, was performed as detailed in the main text. Activity data were collected for $TrpCM^{S220C\text{-}TEMPO}$ (dark green), $ApoCM^{S220C\text{-}TEMPO}$ (black), and $TyrCM^{S220C\text{-}TEMPO}$ (red) under NMR buffer conditions (25 mM $NaHPO_4$, 150 mM NaCl, 1 mM EDTA, 2.8 mM NaN_3, pH 6.5, and 22 °C) following previously established protocols (Sapienza et al. 2021). An additional $TrpCM^{S220C}$ sample without NaCl (light green) was collected to confirm no activity changes under these conditions in order to validate the use of the no-NaCl $TrpCM^{S220C\text{-}TEMPO}$ sample for PRE data collection to optimize NMR peak visualization (see Figure S3). The activity curves for $TrpCM^{S220C\text{-}TEMPO}$, with and without NaCl, align closely with wild-type reports, as previously shown by us and others (Schnappauf et al. 1997; Schnappauf et al. 1998; Helmstaedt et al. 2002; Gorman and Boehr 2019; Sapienza et al. 2021). Notably, apo- and $TyrCM^{S220C\text{-}TEMPO}$ exhibit

hyperbolic activity curves, whereas the wild-type forms exhibit sigmoidal-shaped curves (Schnappauf et al. 1997; Schnappauf et al. 1998; Helmstaedt et al. 2002; Gorman and Boehr 2019; Sapienza et al. 2021). This shift does not change the overall conclusion of this article, as Trp continues to activate the protein, while Tyr inhibits its activity. These results are further supported by the K_m and k_{cat} values presented in Table S2.

CM Form	K_m (mM)	k_{cat} (s^{-1})
$TrpCM^{S220C\text{-}TEMPO}$	3.7 ± 1.9	389.6 ± 35.2
[a]$TrpCM^{S220C\text{-}TEMPO}$	2.3 ± 0.8	297.2 ± 15.2
$apoCM^{S220C\text{-}TEMPO}$	6.8 ± 1.6	345.6 ± 16.6
$TyrCM^{S220C\text{-}TEMPO}$	9.2 ± 1.9	212.3 ± 9.9

[a]Collected without NaCl to mimic NMR experiment conditions. See Figure S3 for more details.

Table S2. Michaelis-Menten Activity Parameters for CM^{S220C}. Trp-bound results are comparable with previously reported wild-type measurements (Schnappauf et al. 1997; Schnappauf et al. 1998; Helmstaedt et al. 2002; Gorman and Boehr 2019; Sapienza et al. 2021). The apo and Tyr-bound forms exhibit lower activity levels compared to the Trp-bound form, maintaining Trp's activator effect as observed in the wild-type protein.

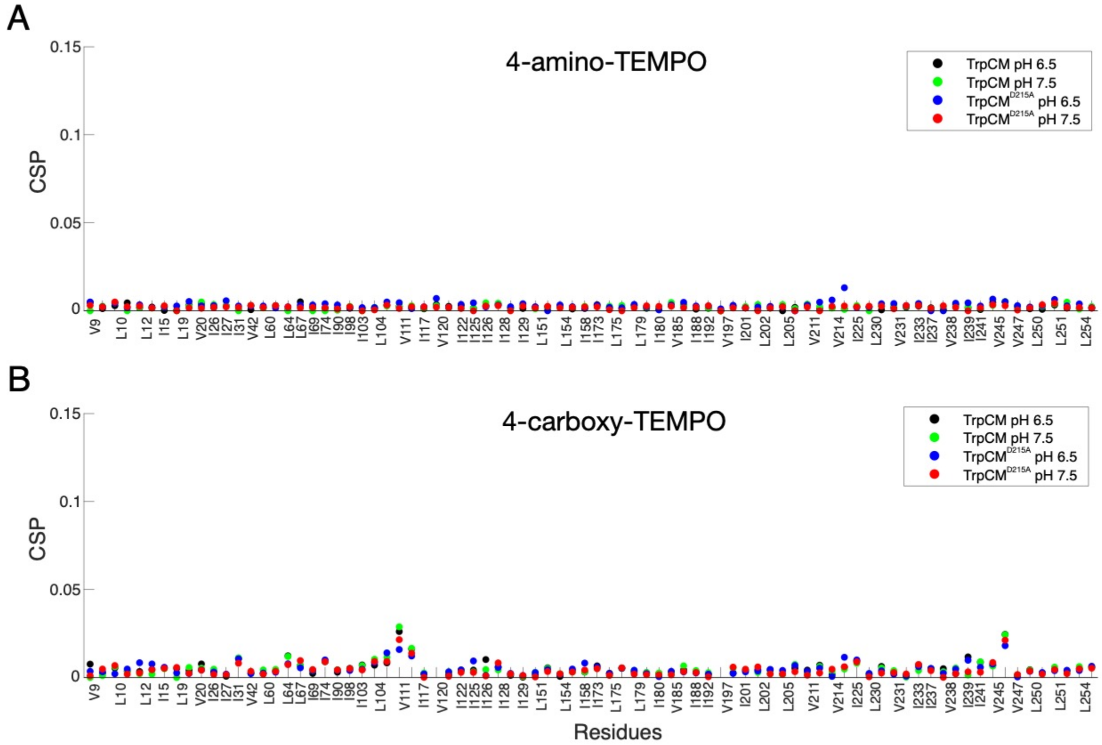

Figure S6. Chemical shift perturbations (CSPs) for (A) 4-amino-TEMPO (4AT) and (B) 4-carboxy-TEMPO (4CT) containing samples. These CSPs were derived from the same spectra used to calculate net charge information presented in Figure 5B of the main text by comparing the spectra of each paramagnetic sample to its respective diamagnetic control. Small, but noticeable CSPs are seen for residues V111 and V245, which are structurally close to each other, in the presence of 4CT. In contrast, 4AT-containing samples exhibited minimal to none CSPs. Any observed CSPs were distant from regions showing significant PRE effects, and therefore do not impact the main conclusions of this study.

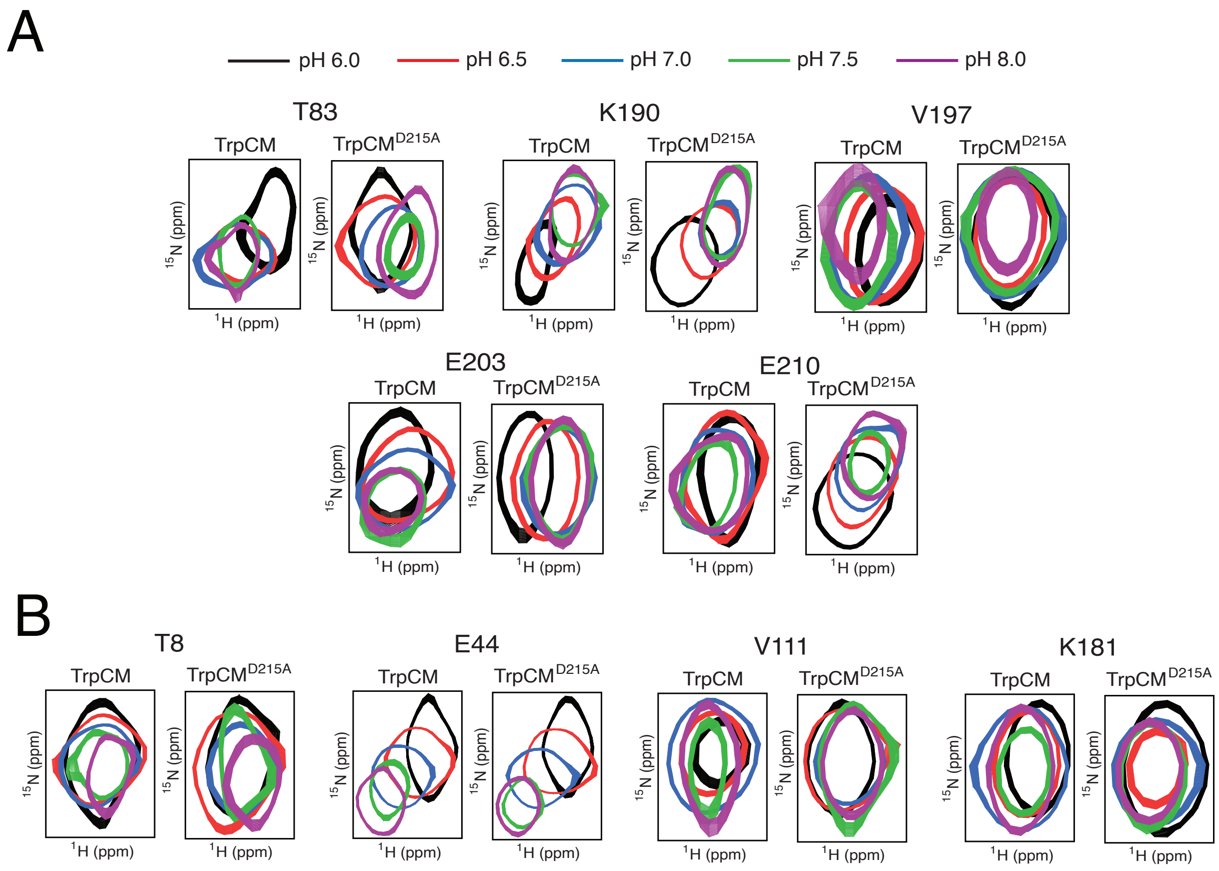

Figure S7. Amide backbone pH titration for TrpCM and TrpCM^{D215A} reveals chemical shift differences between wild-type and mutant forms. ^{1}H-^{15}N HSQC spectra were collected for TrpCM and TrpCM^{D215A} at pH 6.0 (black), 6.5 (red), 7.0 (blue), 7.5 (green), and 8.0 (purple) to monitor pH-dependent chemical shift changes. (A) Residues exhibiting apparent differences in chemical shift behavior between the wild-type and mutant. Residue T83 showed a larger chemical shift between pH 6.0 and 6.5 in the wild-type compared to the mutant, suggesting distinct local charged environments influenced by loop 11-12. T83 is located near the effector binding pocket where tryptophan binds, more than 20 Å from the loop 11-12 stubs. V197, one of the main probes exhibiting differential PREs (Figure 3B and 3C of the main text), also showed distinct titration behavior between TrpCM and TrpCM^{D215A}. Residues K190, E203, and E210, which are located within or near regions with significant differences in effective net charge as

identified by the sPRE experiments (Figure 5 of the main text), also showed apparent chemical shift differences (see also Figure S8). (B) Representative residues that either did not exhibit apparent chemical shift changes across pH values or showed almost identical titration behavior in both the wild-type and mutant, serving as internal controls for regions unaffected by the D215A mutation.

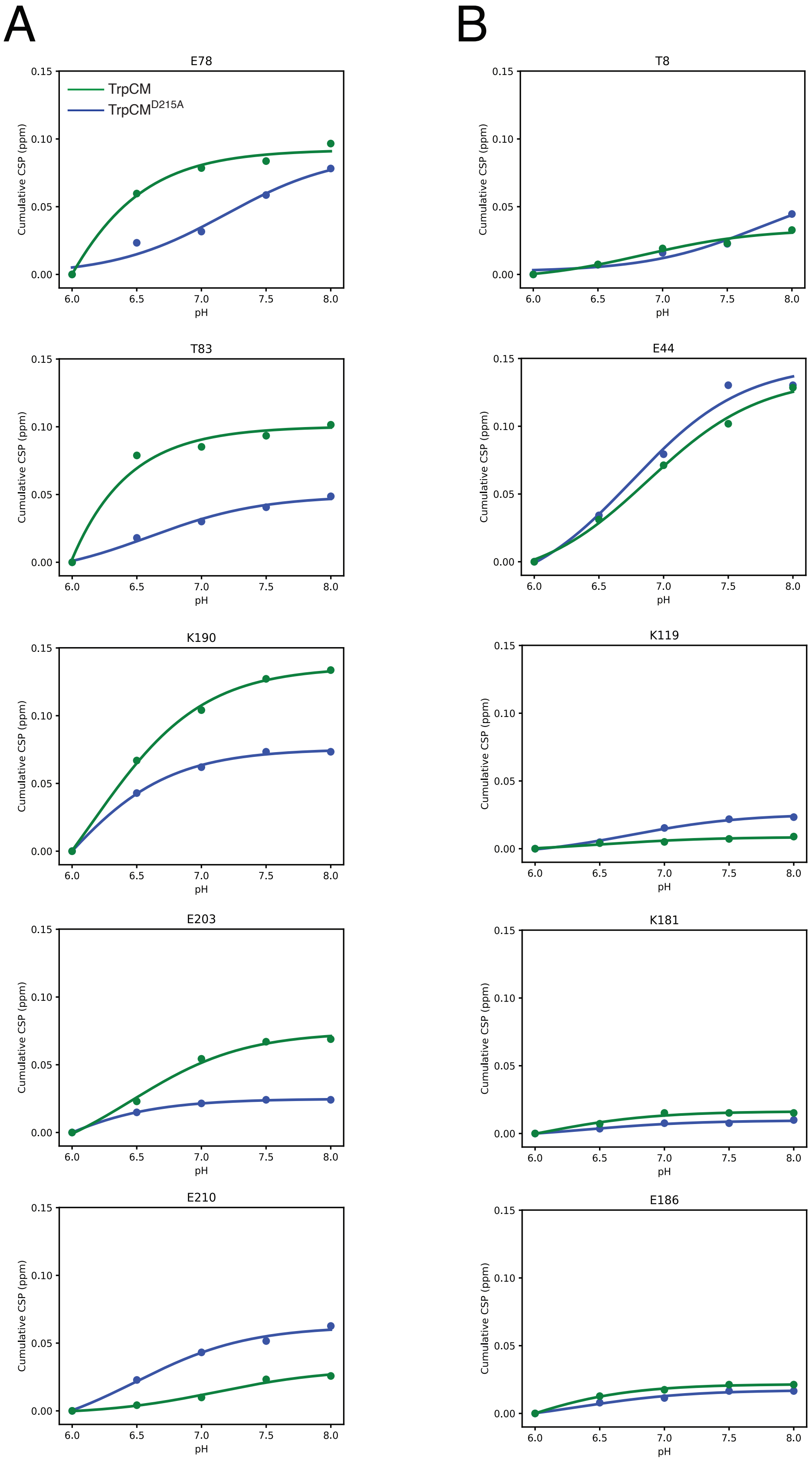
A
B
E78
TrpCM
TrpCMD215A
T83
K190
E203
E210
T8
E44
K119
K181
E186
Cumulative CSP (ppm)
pH
0.15
0.10
0.05
0.00
6.0
6.5
7.0
7.5
8.0

Figure S8. pH titration curves of TrpCM and TrpCM^{D215A}. (A) Titration curves for selected residues showing differences between wild type (green) and the D215A mutant (blue). Residues E78 and T83 are located near the tryptophan binding pocket, K190 and E203 are located near the active site, and E210 is close to loop 11-12 (residues 212-226). These residues were chosen based on their relative differences in cumulative chemical shift perturbations (CSPs) between CM forms (see Methods below), as well as the ability to track the chemical shifts across pH spectra. Residues shown are representative of the main regions exhibiting differences in titration behavior between TrpCM and TrpCM^{D215A}. (B) Residues showing similar titration curves for TrpCM and TrpCM^{D215A}. The majority of traceable residues in this experiment fell into this category (i.e., no major differences in titration curves). Five residues are shown as reference for internal controls.

METHODS

NMR pH Titration Acquisition, Processing, and Analysis. Five samples each for TrpCM and $TrpCM^{D215A}$ were prepared at pH values of 6.0, 6.5, 7.0, 7.5, and 8.0. Samples were loaded into 5 mm NMR tubes, and ^{1}H-^{15}N HSQC spectra were recorded on a Bruker Avance III HD 850 MHz spectrometer at 30 °C. NMRPipe (Delaglio et al. 1995) and NMR View (Johnson and Blevins 1994) were used for data processing and analysis. Amide CSPs were calculated as follows:

$$CSP = \sqrt{\left(\Delta\,^{1}H\right)^{2} + 0.14\left(\Delta^{15}N\right)^{2}}$$

where 0.14 is the amide chemical shift scaling factor as previously defined (Williamson 2013). Data points to generate the titration curve were calculated using the equation:

$$Cumulatitive\ CSP = CSP_{i-1} + CSP_{i}$$

where *i* is the current data point. The pH 6.0 data point in the plots was set to 0 as the initial reference. The Henderson-Hasselbalch equation was used to fit the data to provide a visual interpretation of the titration behavior. However, pKa values could not be accurately determined due to the absence of chemical shifts measurements below pH 6.0 and above pH 8.0.

REFERENCES

Sapienza PJ et al. 2021. Visualizing an Allosteric Intermediate Using CuAAC Stabilization of an NMR Mixed Labeled Dimer. ACS Chemical Biology. 2021. https://doi.org/10.1021/acschembio.1c00617

Sapienza PJ et al. 2023. Mixed, nonclassical behavior in a classic allosteric protein. Proceedings of the National Academy of Sciences. 2023;120(38):e2308338120. https://doi.org/10.1073/pnas.2308338120

Gorman SD, Boehr DD. 2019. Energy and Enzyme Activity Landscapes of Yeast Chorismate Mutase at Cellular Concentrations of Allosteric Effectors. Biochemistry. 2019;58(39):4058–4069. https://doi.org/10.1021/acs.biochem.9b00721

Helmstaedt K, Heinrich G, Lipscomb WN, Braus GH. 2002. Refined molecular hinge between allosteric and catalytic domain determines allosteric regulation and stability of fungal chorismate mutase. Proceedings of the National Academy of Sciences. 2002;99(10):6631–6636. https://doi.org/10.1073/pnas.092130899

Schnappauf G, Sträter N, Lipscomb WN, Braus GH. 1997. A glutamate residue in the catalytic center of the yeast chorismate mutase restricts enzyme activity to acidic conditions. Proceedings of the National Academy of Sciences. 1997;94(16):8491–8496. https://doi.org/10.1073/pnas.94.16.8491

Schnappauf G, Lipscomb WN, Braus GH. 1998. Separation of inhibition and activation of the allosteric yeast chorismate mutase. Proceedings of the National Academy of Sciences. 1998;95(6):2868–2873. https://doi.org/10.1073/pnas.95.6.2868

Delaglio F et al. 1995. NMRPipe: A multidimensional spectral processing system based on UNIX pipes. Journal of Biomolecular NMR. 1995;6(3):277–293. https://doi.org/10.1007/bf00197809

Johnson BA, Blevins RA. 1994. NMR View: A computer program for the visualization and analysis of NMR data. Journal of Biomolecular NMR. 1994;4(5):603–614. https://doi.org/10.1007/bf00404272

Williamson MP. 2013. Using chemical shift perturbation to characterise ligand binding. Progress in Nuclear Magnetic Resonance Spectroscopy. 2013;73:1–16. https://doi.org/10.1016/j.pnmrs.2013.02.001
